# Comparison of SARS-CoV-2 sequencing using the ONT GridION and the Illumina MiSeq

**DOI:** 10.1186/s12864-022-08541-5

**Published:** 2022-04-22

**Authors:** Derek Tshiabuila, Jennifer Giandhari, Sureshnee Pillay, Upasana Ramphal, Yajna Ramphal, Arisha Maharaj, Ugochukwu Jacob Anyaneji, Yeshnee Naidoo, Houriiyah Tegally, Emmanuel James San, Eduan Wilkinson, Richard J. Lessells, Tulio de Oliveira

**Affiliations:** 1 KwaZulu-Natal Research Innovation and Sequencing Platform (KRISP), School of Laboratory Medicine & Medical Sciences, University of KwaZulu-Natal Durban 4001, KwaZulu-Natal, South Africa; 2 Centre for Epidemic Response and Innovation (CERI), Stellenbosch University, Stellenbosch, South Africa; 3 Centre for the AIDS Programme of Research in South Africa (CAPRISA), Durban, South Africa; 4 Department of Global Health, University of Washington, Seattle, WA USA

**Keywords:** SARS-CoV-2, Illumina MiSeq, Oxford Nanopore technology GridION, Nanopore sequencing, next generation sequencing, bioinformatics

## Abstract

**Background:**

Over 4 million SARS-CoV-2 genomes have been sequenced globally in the past 2 years. This has been crucial in elucidating transmission chains within communities, the development of new diagnostic methods, vaccines, and antivirals. Although several sequencing technologies have been employed, Illumina and Oxford Nanopore remain the two most commonly used platforms. The sequence quality between these two platforms warrants a comparison of the genomes produced by the two technologies. Here, we compared the SARS-CoV-2 consensus genomes obtained from the Oxford Nanopore Technology GridION and the Illumina MiSeq for 28 sequencing runs.

**Results:**

Our results show that the MiSeq had a significantly higher number of consensus genomes classified by Nextclade as good and mediocre compared to the GridION. The MiSeq also had a significantly higher genome coverage and mutation counts than the GridION.

**Conclusion:**

Due to the low genome coverage, high number of indels, and sensitivity to SARS-CoV-2 viral load noted with the GridION when compared to MiSeq, we can conclude that the MiSeq is more favourable for SARS-CoV-2 genomic surveillance, as successful genomic surveillance is dependent on high quality, near-whole consensus genomes.

## Background

December 2019 saw a novel viral pneumonia emerge from a seafood market in Wuhan China later found to be a new type of Coronavirus, now known as Severe Acute Respiratory Syndrome Coronavirus 2 (SARS-CoV-2) [1, 2]. On 11 March 2020, after approximately 118,000 cases had been reported globally, the World Health Organization (WHO) declared SARS-CoV-2 a global pandemic [3, 4]. SARS-CoV-2 is an ongoing pandemic that requires continuous surveillance with approximately 270,031,622 cases confirmed globally as of 14 December 2021 [3, 5].

Sequencing of SARS-CoV-2 allowed for the rapid identification of the virus and the development of diagnostic tests and other tools for a rapid response to the pandemic [6]. Sequencing provides genotypic information about a patient’s infection, which can be used to gain knowledge on the specific infecting strain, assist in identifying transmission within communities, and advance the development of new diagnostic methods, vaccines, and antivirals [7]. Multiple next generation sequencing (NGS) technologies have been used for SARS-CoV-2 sequencing, including Sanger, Illumina, ION torrent, and Oxford Nanopore Technology [8]. However, Illumina sequencing remains the most commonly used technology [9]. As of 05 November 2021, 4,892,742 SARS-CoV-2 consensus genomes had been deposited into the Global Initiative on Sharing all Influenza Data (GISAID) with over 65% from Illumina and approximately 25% from Oxford Nanopore Technology (ONT) [10].

A major challenge with whole-genome sequencing (WGS) is obtaining whole viral genomes from clinical samples promptly [11]. Illumina SARS-CoV-2 sequencing is generally limited by long sequencing times and the high cost and labour associated with library preparation for high-throughput sequencing [12]. Another limitation is their relatively short reads (2 × 300 bp), as genomes generally contain multiple repeated sequences, known as tandem repeats, that may be longer than the NGS reads and may result in gaps and misassemblies [13]. Due to the large footprint of most sequencers, portability can be a challenge which is unfortunate as there is generally a large distance between sample collection sites and sequencing laboratories [14]. Nanopore sequencing overcomes these challenges as they sequence in real-time and are long-read sequencing technologies that allow for portability and have a relatively low initial investment on sequencing equipment with the MinION costing $1000 [15]. ONT sequencing is, however, limited by the high number of false negatives and low sensitivity [16].

Short-read sequencing technologies are useful for population-level genetic analysis and clinical variant discovery as they provide low-cost, high-accuracy data when done in large batches. Long-read sequencing approaches, however, are well suited for de novo genome assembly, sequencing of genomes with long repetitive regions, copy number alterations, and complex structural variations [17]. Several studies have compared the sequencing of SARS-CoV-2 between Illumina and ONT platforms and have shown that despite the high error rates observed with ONT sequencing, highly-accurate SARS-CoV-2 consensus genomes can be achieved [18]. ONT sequencing, however, failed to detect short indels identified by Illumina sequencing [18]. There has also been a lower raw-read accuracy with nanopore sequencing when compared to Illumina sequencing [18, 19].

A comparison of SARS-CoV-2 WGS genomic coverage and variant detection between Illumina and Nanopore sequencing is necessary as it allows us to determine whether SARS-CoV-2 genomes produced by Nanopore sequencing can be reliably used for genomic surveillance and the development of diagnostic measures. As SARS-CoV-2 lineages differ by geographic location, this study aimed to determine whether Nanopore sequencing is a viable alternative to Illumina sequencing for rapidly identifying SARS-CoV-2 variants found within African countries. We hypothesize that Nanopore sequencing will produce consensus genomes that are comparable to consensus genomes produced by Illumina sequencing at a faster rate. SARS-CoV-2 sequencing results, for multiple runs, from the Illumina MiSeq and the ONT GridION were compared and although Nanopore sequencing was able to produce complete SARS-CoV-2 genomes, the quality observed was not as good as those obtained with Illumina sequencing. The ONT GridION can sequence up to 5 flowcells with 96 samples in a single run and is cheaper than sequencing with the Illumina MiSeq. These advantages can allow for more clinical facilities to sequence SARS-CoV-2 allowing for a greater response to the COVID-19 pandemic.

## Results

### Comparison of sequencing performance

**Fig. 1:**
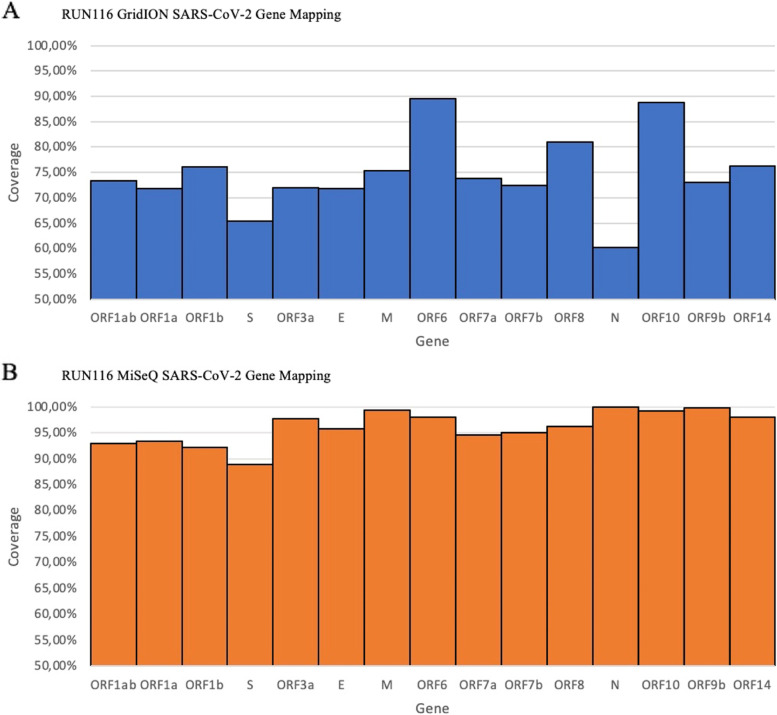
Comparison of GridION and MiSeq gene mapping for RUN116: Sequencing files from both the Illumina MiSeq and the ONT GridION were assembled using Genome Detective and average coverage across the 15 known genes was calculated to determine the sequencing coverage across the genome

To compare sequencing performance and runtime between the MiSeq and the GridION, Run116 was sequenced on both platforms (Table 1). A total of 93 samples were sequenced and 93 consensus genomes were produced after assembly using Genome Detective. The sequencing runtime for the MiSeq was 36 h, whilst the GridION had a runtime of 21 h. The MiSeq had an overall higher average coverage than the GridION, having coverages of 94.34 and 72.96%, respectively. There was also a higher number of consensus genomes that passed the QC used for GISAID submissions (> 80% genome coverage) from the MiSeq, 83 (89.2%), than the GridION, 29 (27.9%). The average coverage across the genome for the GridION (Fig. 1-A) was less uniform than that of the MiSeq (Fig. 1-B).

**Table 1:** Comparison of sequencing Run116 on both the MiSeq and the GridION

	Run116	
	MiSeq	GridION
Runtime (hrs.)	36	21
No. of samples sequenced	93	93
Data obtained	7246.3 MB	1999,4 MB
Consensus genomes	93	93
Average coverage (X)	94.34%	72.96%
Passing GISAID QC (> + 80%)	83 (89.2%)	29 (27.9%)
Clusters passing QC	70%	–
Q30 score	73.1%	–
Pores on flowcell	–	1012 pores

The table above summarizes the sequencing of Run116 on both the MiSeq and the GridION. The sequencing runtime for the MiSeq was 36 h, whilst that of the GridION was 21 h. The MiSeq had a Q30 score of 73.1% with 70% of the clusters passeing QC The flowcell used for the GridION had 1012 pores available for sequencing. Of the 93 samples sequenced by both platforms, 93 consensus genomes were produced by each. Consensus genomes from the MiSeq had an average coverage of 94.34% with 89.2% having a coverage of 80% and over. Consensus genomes from the GridION had an average coverage of 72.96% with 27.9% having a coverage of 80% and over

### Comparison of consensus genome quality of Nanopore and Illumina sequencing

**Fig. 2:**
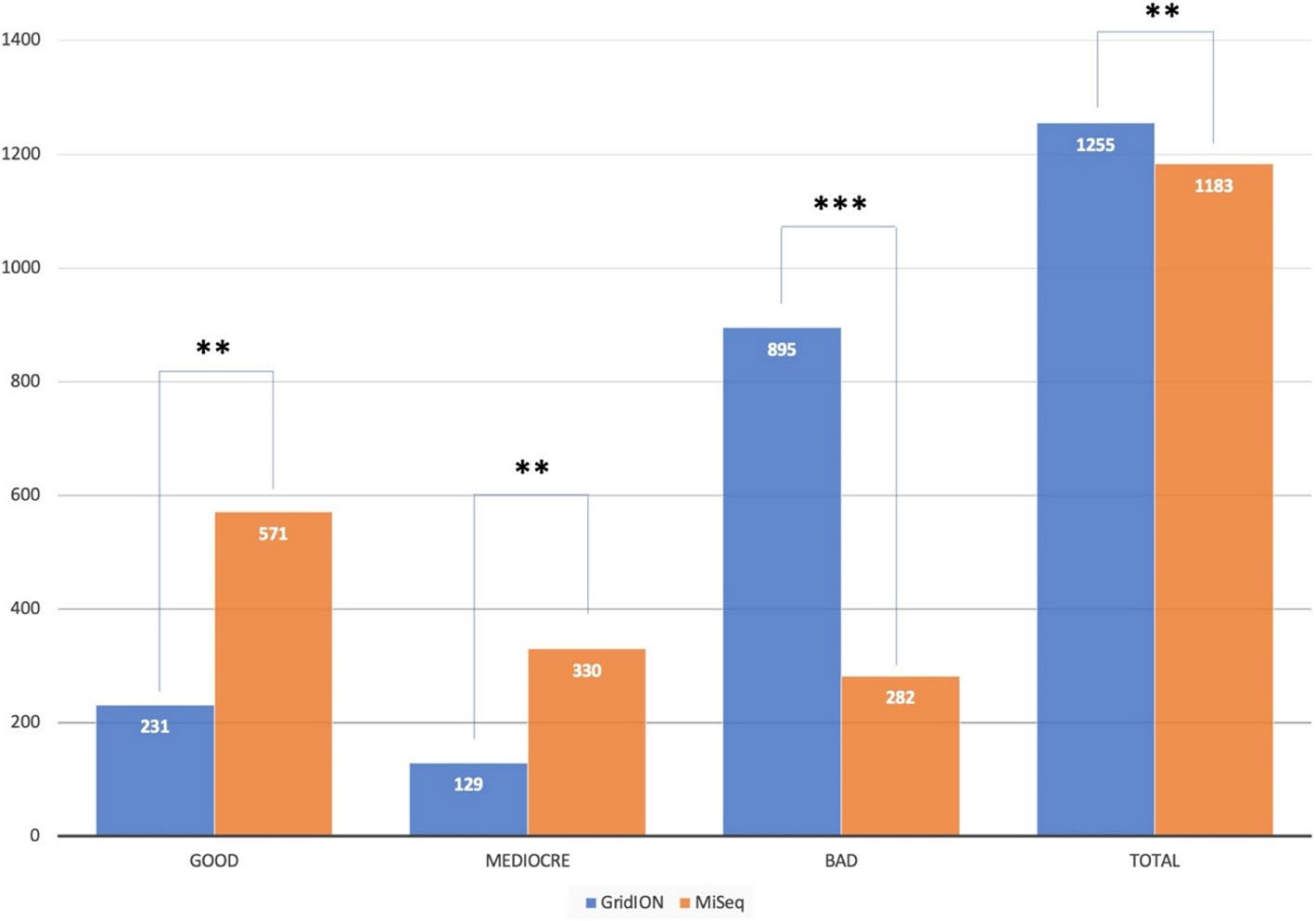
Comparison of consensus genome quality obtained from the GridION and the MiSeq and analyzed on Nextclade: To compare the quality of consensus genomes obtained from the GridION and the MiSeq, consensus genomes from both platforms were uploaded to Nextclade and the results plotted on a double bar graph. Genome quality was broken down into three groups; good, mediocre, and bad, with the GridION represented in blue and the MiSeq represented in orange. Statistical significance (Wilcoxon rank sum tests) is represented by “*” (**: *p <* 0.01, ***: *p <* 0.001). Sequencing scores ranging between 0 and 29 are classified as good, 30 – 99 are classified as mediocre, whilst 100 and above are classified as bad

Consensus genomes produced by the GridION and the MiSeq were uploaded to Nextclade to determine the genome quality. Nextclade classifies genomes as either good, mediocre, or bad, based on the amount of missing data, and the number of mixed sites, private mutations, clustered mutations, frameshifts, and misplaced stop codons. Both the GridION and the MiSeq had a total of 14 runs with 1255 and 1183 consensus genomes, respectively. The total number of consensus genomes produced by the GridION and the MiSeq was significantly different (*p =* 0.0053). The number of genomes the two platforms classified as good (*p =* 0.00280), mediocre (*p =* 0.00250), and bad (*p =* 0.00037) also differed significantly (Fig. 2).

### Comparison of genome coverage generated by the GridION and MiSeq

**Fig. 3:**
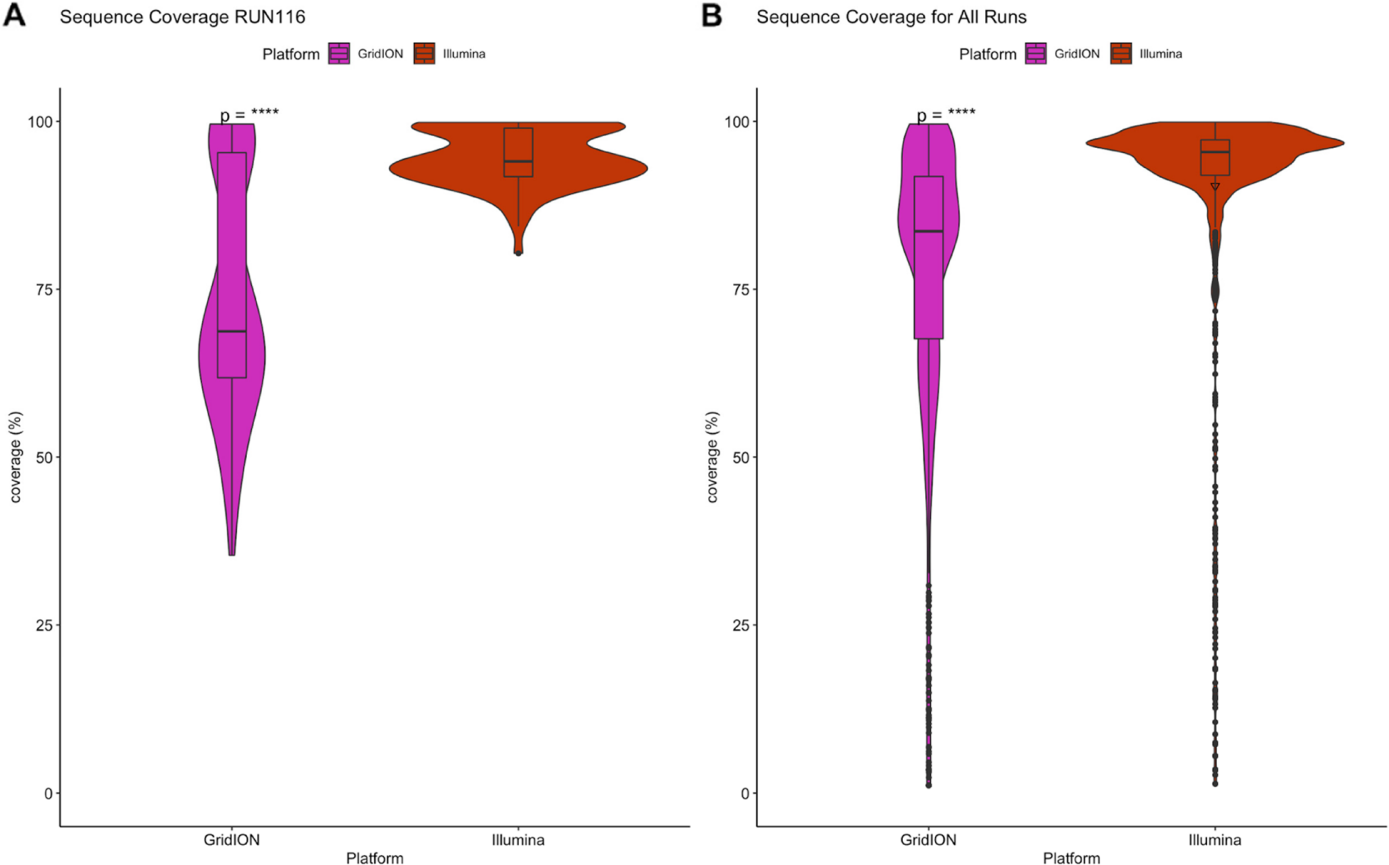
Comparison of GridION and MiSeq genome coverage: Fastq files for RUN116 from both the MiSeq and the GridION were assembled using Genome Detective and the consensus genome coverage was compared (**A**). The same was done for all genomes for both platforms (**B**). GridION samples are presented in purple, whilst Illumina MiSeq samples are presented in red. Statistical significance (Wilcoxon rank sum tests) is represented by “*” (****: *p <* 0.0001)

Identical samples (RUN116) were sequenced on both the GridION and the MiSeq and the genomic coverage was compared to determine the effect of sample quality on sequencing (Fig. 3-A). All the runs for both platforms were then compared (Fig. 3-B). A total of 86 consensus genomes were used from RUN116 after removing genomes with more than 100 mutations. Samples run on the MiSeq had a significantly greater genome coverage than the GridION (*p =* 8.1e-16). GridION genomes ranged from 35 to 100%, whilst MiSeq genomes ranged from 80 to 100%. The consensus genome coverage for all runs, 2351 genomes, was then compared. There was a significantly higher overall genome coverage observed with the MiSeq than with the GridION (*p <* 2.2e-16).

### Comparison of Orf1ab- and S-gene coverage for GridION and MiSeq sequencing

**Fig. 4:**
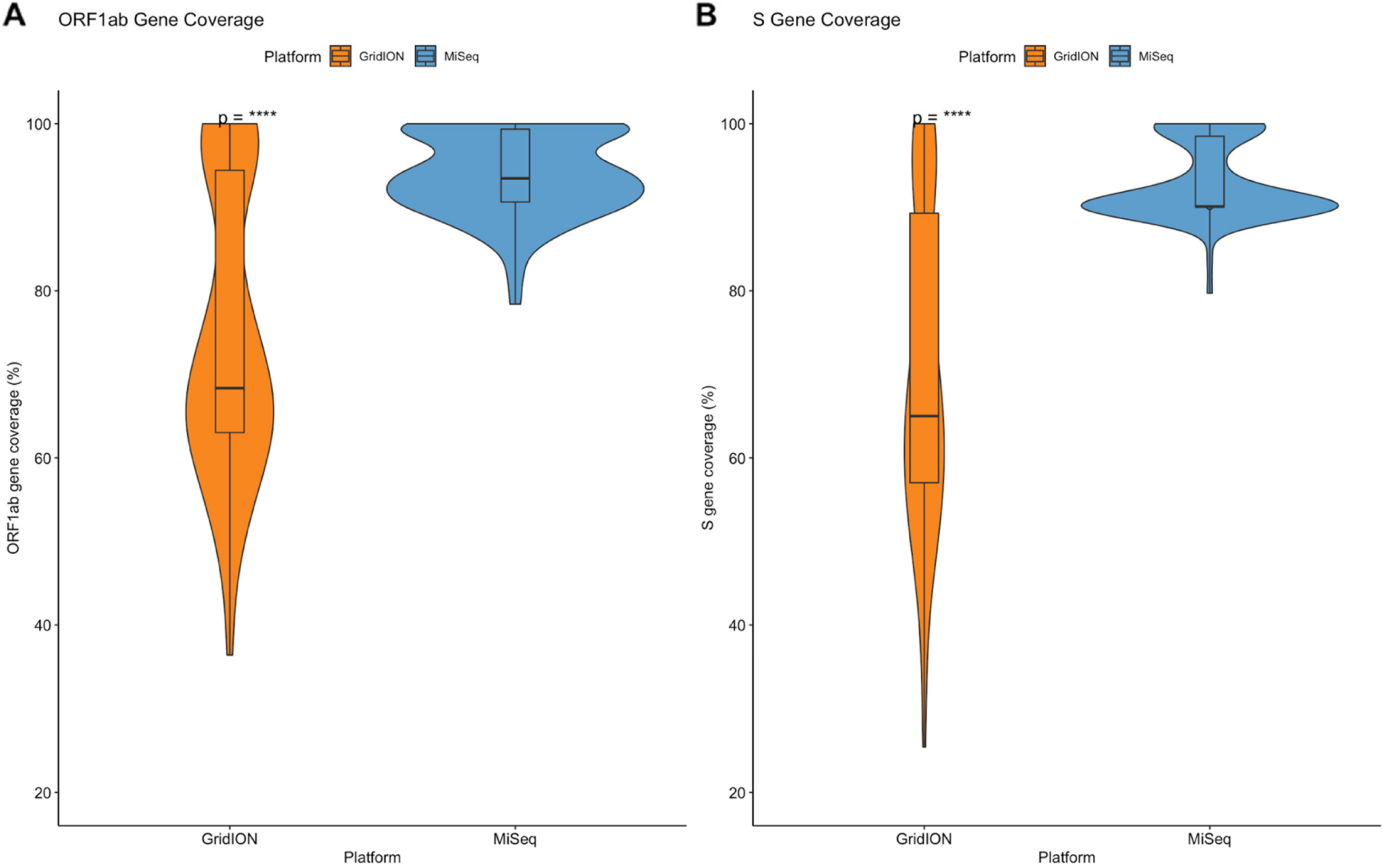
Comparison of ORF1ab- and S-gene coverage on the GridION and the MiSeq: Fastq files produced by both platforms were assembled on Genome Detective and the coverage for the ORF1ab- (A) and S-gene (B) was compared. Consensus genomes from the GridION are represented in orange and genomes from the MiSeq are represented in blue. Statistical significance (Wilcoxon rank sum tests) is represented by “*” (****: *p <* 0.0001)

To compare the depth of coverage of the ORF1ab- and S-gene for the GridION and the MiSeq, fastq files produced from both platforms were assembled on Genome Detective to produce consensus genomes. The results for each consensus genome were obtained and the coverages for the ORF1ab-gene (Fig. 4-A) and S-gene (Fig. 4-B) were compared. All 14 runs for each platform were compared and Wilcoxon rank sum tests were performed. The ORF1ab-gene coverage ranged from 35 to 100% for the GridION and 80 – 100% for the MiSeq. The S-gene coverage ranged from 25 to 100% for the GridION and 80 – 100% for the MiSeq. There was a statistically significant difference in coverage for both genes on the GridION and the MiSeq with *p =* 1.2e-15 (RUN116) and *p =* 1.7e-15 (all genomes).

### Effect of Ct score on sequencing using the GridION and MiSeq

**Fig. 5:**
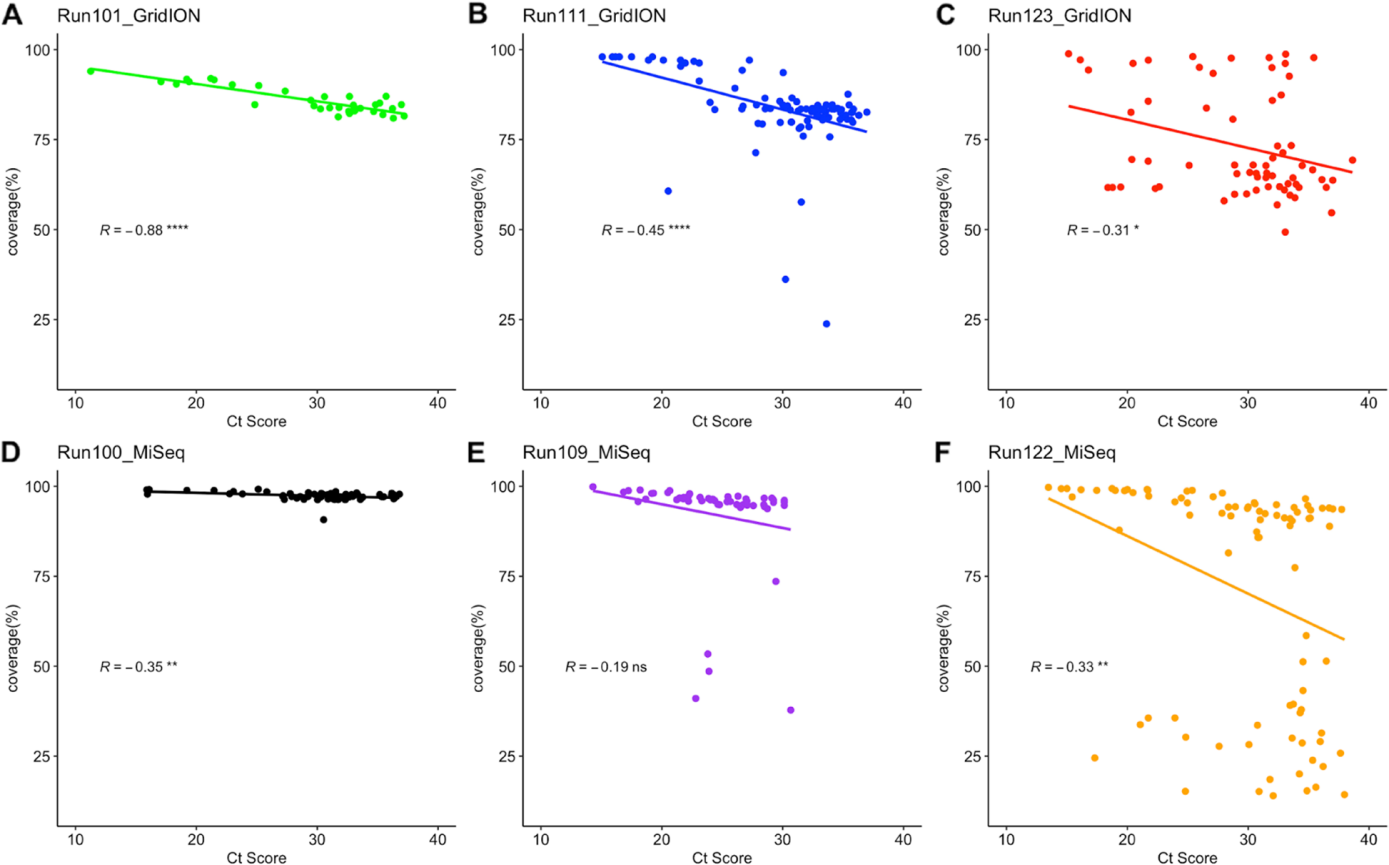
Correlation between genome coverage and Ct score for samples sequenced on the GridION and MiSeq: A correlation was performed to determine the effect of Ct score on the consensus genome coverage obtained from the GridION and the MiSeq. Genome coverage was plotted on the y-axis, whilst the sample’s average Ct score was plotted on the X-axis. GridION runs are represented by graphs **A** (Run101), **B** (Run111), and **C** (Run123), which are represented as green, blue, and red, respectively. MiSeq runs are represented by graphs **D** (Run100), **E** (Run109), and **F** (Run122) and are represented as black, purple, and gold, respectively. Statistical significance (Spearman’s rank correlation test) is represented by “*” (ns: non-significant, *: *p <* 0.05, **: *p <* 0.01, ***: *p <* 0.001, ****: *p <* 0.0001). For both platforms, as the Ct score increased, there was a decrease in genomic coverage

**Fig. 6:**
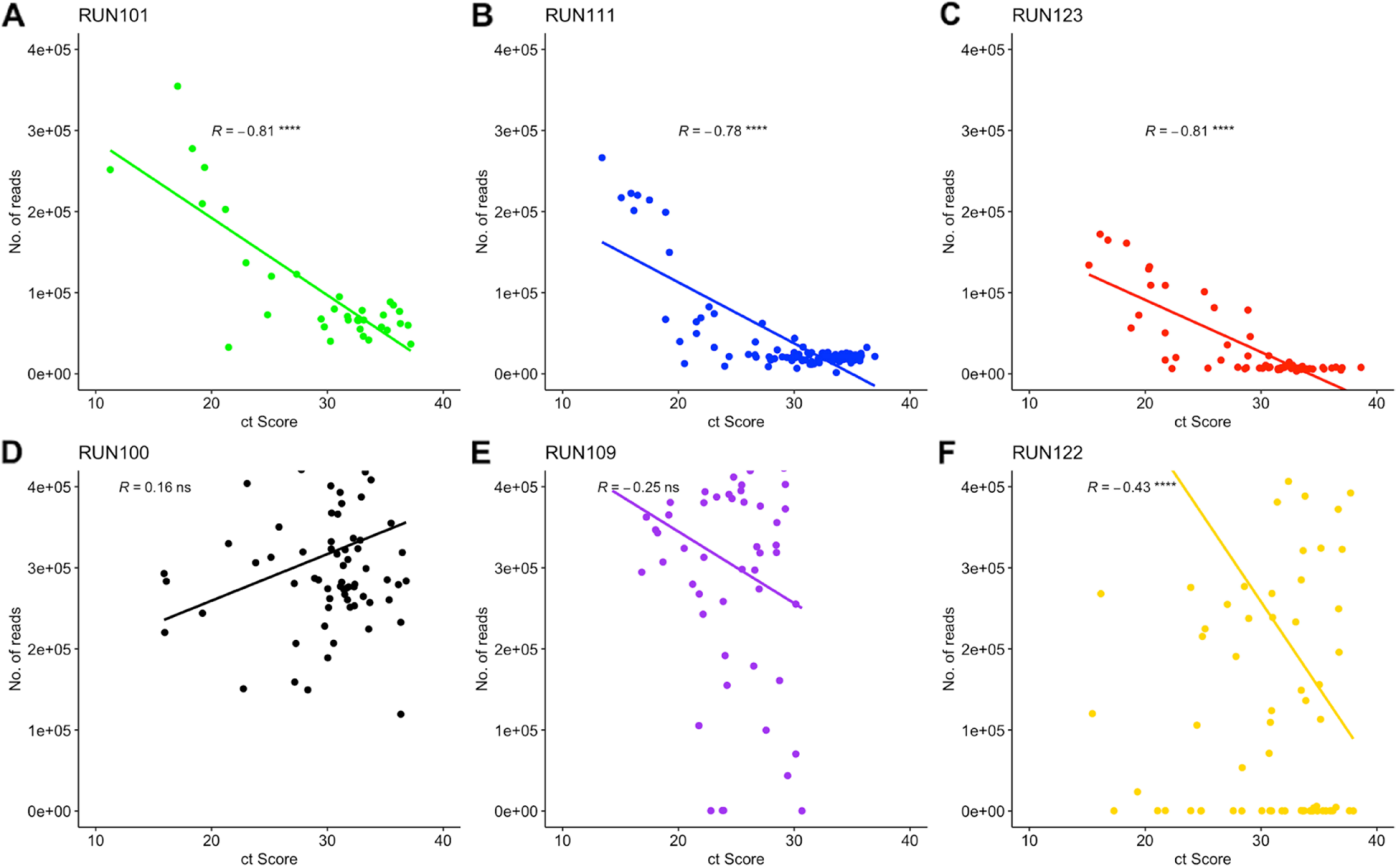
Correlation between the number of reads produced during sequencing and sample Ct Score: A correlation was performed for the number of reads produced by the GridION and the MiSeq and Ct score for SARS-CoV-2 samples. The number of reads was plotted on the Y-axis, whilst each sample’s average Ct score was plotted on the X-axis. GridION runs are represented by graphs **A** (Run101), **B** (Run111), and **C** (Run123) and are shown as green, blue, and red, respectively. MiSeq runs are represented by graphs **D** (Run100), **E** (Run109), and **F** (Run122) and are shown as black, purple, and gold, respectively. Statistical significance (Spearman’s rank correlation test) is represented by “*” (ns: non-significant, ****: *p <* 0.0001). An increase in Ct score resulted in a decrease in the number of reads produced for all GridION runs and 1 Illumina MiSeq run (Run122)

A correlation was performed to determine the effect of Ct score on genome coverage (Fig. 5) and the number of reads produced by the GridION and the MiSeq during sequencing (Fig. 6). Due to the availability of Ct scores, three runs were used for each platform. Run101 (35 samples), Run111 (91 samples), and Run123 (64 samples), represented by graphs A, B, and C, respectively, were used for the GridION. Run100 (68 samples), Run109 (54 samples), and Run122 (88 samples), represented by graphs D, E, and F, respectively, were used for the MiSeq. A negative correlation was observed between Ct Score and genome coverage for all six runs. The GridION’s Runs 101, 111, and 123 had correlation coefficients of *R =* − 0.88 (*p =* 4.5e-12), *R =* − 0.45 (*p =* 7.2e-06), and *R =* − 0.31(*p =* 0.012), respectively. The MiSeq’s Runs 100, 109, and 122 had correlation coefficients of *R =* − 0.35 (*p =* 0.0039), *R =* − 0.19 (*p =* 0.18), and *R =* − 0.33 (*p =* 0.0017), respectively. We note a significantly strong negative correlation between Ct score and number of reads for all GridION runs, whereas a significantly negative correlation was only noted for Run122 sequenced on the MiSeq. Run100 and Run109 showed non-significant correlations.

### Mutation analysis

**Fig. 7:**
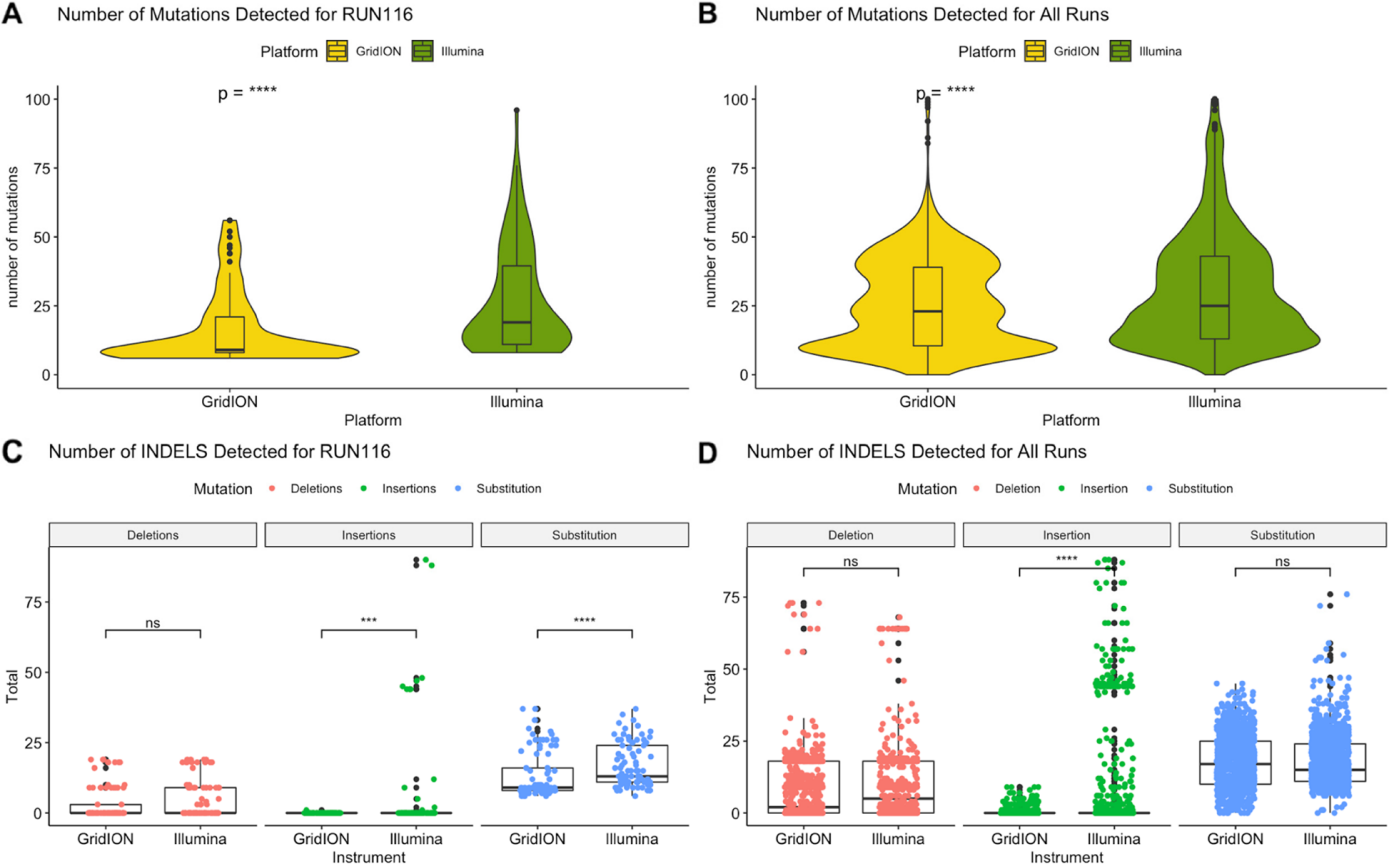
Analysis of mutations in samples sequenced on the GridION and the MiSeq: Consensus genomes produced by Genome Detective were uploaded to Nextclade and the results were analyzed. RUN116 was run on both platforms and the number and type of mutations detected by each platform was compared using a Wilcoxon rank sum test (Fig. **A** and **C**). A consensus file for all runs, for each platform, was produced and uploaded to Nextclade and a Wilcoxon rank sum test was performed to compare the number and type of mutations detected by both platforms (Fig. **B** and **D**). GridION samples are represented in yellow, whilst MiSeq samples are presented in green. Deletions, insertions, and substitutions are represented in pink, green, and blue, respectively. Statistical significance (Wilcoxon p tests) is represented by “*” (ns: non-significant, ***: *p <* 0.001, ****: *p <* 0.0001)

To determine whether the number of mutations detected by GridION and MiSeq differed significantly, the number of mutations detected for each sample was compared for Run116 (Fig. 7-A) and all the runs (Fig. 7-B). The total number of insertions, deletions, and substitutions detected by both platforms were also compared for Run116 (Fig. 7-C) and all the runs (Fig. 7-D). A total of 181 consensus genomes obtained from the GridION and the MiSeq for Run116 were analyzed and a significant difference was noted in the number of mutations detected by each platform (Wilcoxon, *p =* 3.7e-08) with a greater number of mutations detected by the MiSeq (8 – 96 mutations) than the GridION (6 – 56 mutations). We also noted a significant difference (Wilcoxon, *p =* 1.5e-09) between the number of mutations detected from the genomes obtained from the MiSeq (1183 genomes) and the GridION (1255 genomes). There was a significant difference in the number of insertions (Wilcoxon, *p =* 8.2e-04) and substitutions (Wilcoxon, *p =* 5.3e-06) detected by both platforms for RUN116. However, when all runs were analyzed; only the number of insertions were significantly different between the two platforms (Wilcoxon, *p =* 7.5e-15).

### Phylogenetic analysis

**Fig. 8:**
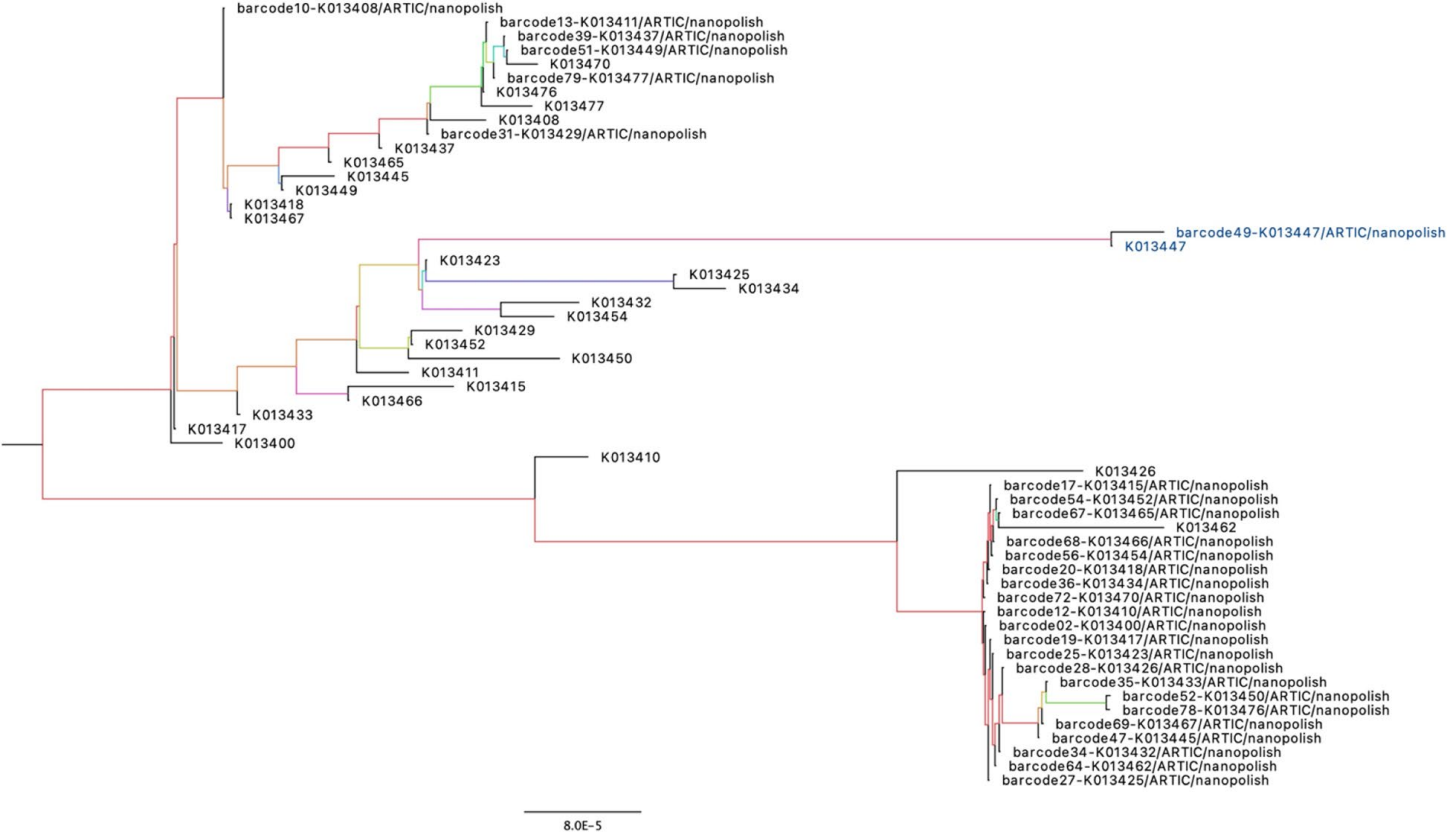
Phylogenetic comparison between identical samples sequenced using both the GridION and MiSeq: A phylogenetic tree was created using IQTREE and visualized using FigTree for samples from Run116 sequenced on both the GridION and the MiSeq but classified in different clades by Nextclade. Only one of the 27 samples, represented in blue, clustered on the same branch. GridION genomes are annotated as ‘barcode*’, whilst MiSeq genomes are annotated as ‘K0*’

To determine whether there was a difference in the phylogenetic inference between consensus genomes generated by the GridION and the MiSeq, Run116 samples were sequenced on both platforms. A total of 93 consensus genomes from both the GridION and the MiSeq were uploaded to Nextclade and the results were compared. Of the 93 samples, 27 samples were classified within different clades (Table 2). A phylogenetic tree of the 27 samples was then created using IQTREE and visualized using FigTree (Fig. 8). Of the 27 samples, only one sample, highlighted in blue, was grouped on the same branch.

**Table 2:** Comparison of the genome coverage and assigned clade for run116 samples on Nextclade

	Coverage (%)		Clade	
Sample	**GridION**	**MiSeq**	**GridION**	**MiSeq**
K013400	72	92	20C	20A
K013408	57	86	20H (Beta, V2)	20C
K013410	70	90	20C	20A
K013411	76	93	20H (Beta, V2)	20A
K013415	63	91	20C	20A
K013417	63	94	20C	20A
K013418	62	94	20C	20A
K013423	63	91	20C	20A
K013425	60	93	20C	20A
K013426	57	89	20C	20A
K013429	64	95	20H (Beta, V2)	20C
K013432	65	93	20C	20A
K013433	50	94	20C	20A
K013434	68	94	20C	20A
K013437	35	92	20H (Beta, V2)	20C
K013445	65	91	20C	20A
K013447	92	98	20A	20D
K013449	49	94	20C	20A
K013450	68	97	20C	20A
K013452	68	94	20C	20A
K013454	56	90	20C	20A
K013462	51	92	20C	20A
K013465	60	94	20C	20A
K013467	50	92	20C	20A
K013470	72	89	20C	20H (Beta, V2)
K013476	69	91	20C	20H (Beta, V2)
Total	20A		1	20
	20C		22	3
	20D		0	1
	20H (Beta, V2)		4	3

The table above highlights the 27 samples which were sequenced on both the MiSeq and the GridION but were classified in different clades by Nexclade. Clades identified by the GridION include 20A (*n* = 1), 20C (*n* = 22), and 20H (Beta, V2) (*n* = 4). Clades identified by the MiSeq include 20A (*n* = 20), 20C (*n* = 3), 20D (*n* = 1), and 20H (Beta, V2) (*n* = 3). There was also an overall higher genomic coverage for sequences from the MiSeq when compared to the GridION.

The table above highlights the 27 samples which were sequenced on both the MiSeq and the GridION but were classified in different clades by Nexclade. Clades identified by the GridION include 20A (*n =* 1), 20C (*n =* 22), and 20H (Beta, V2) (*n =* 4). Clades identified by the MiSeq include 20A (*n =* 20), 20C (*n =* 3), 20D (*n =* 1), and 20H (Beta, V2) (*n =* 3). There was also an overall higher genomic coverage for sequences from the MiSeq when compared to the GridION.

## Discussion

SARS-CoV-2 has caused a global health crisis as it is highly infectious and risks mutations that could result in more lethal variants [1, 20]. A major factor in helping curb the spread of the virus and decreasing the infection rate is rapidly sequencing the virus to detect new strains and identify transmission chains [7]. The sequencing runtime on the MiSeq for Run116 was 36 h, whilst on the GridION it was 21 h. This 10-h decrease in sequencing time allows for 480 samples to be sequenced each day on the GridION in comparison to the 96 that can be sequenced on the MiSeq every 36 h. This is in agreement with reports that nanopore sequencing takes approximately 20 h as a rapid library prep kit supplied by ONT can be used [21, 22]. The lack of an image analysis step during nanopore sequencing facilitates real-time base-calling, which allows for the rapid detection of DNA for pathogen screening from clinical samples [23].

Studies have shown that Illumina sequencing may still be the most accurate way to sequence viruses [24]. The majority of errors noted between Nanopore and Illumina consensus genomes have been attributed to Nanopore sequencing errors [25]. Run116 samples were sequenced on both platforms to determine whether there was a significant difference in the sequencing coverage regardless of the sample. Consensus genome coverage was significantly greater with the MiSeq when compared to the GridION and this result was also observed when comparing all sequence runs. Genomic coverage can be affected by sequencing time and thus GridION coverage may have increased if left to sequence for longer. We also note a statistically significant higher sequencing coverage for the S-gene and ORF1ab-gene with the MiSeq than with the GridION. Nanopore technology has been shown to provide lower per-read sequencing coverage when compared to short-read sequencing [26]. Coverage biases seen with ONT’s sequencing protocol can be a result of truncated reads caused by pore blocking or fragmentation during library prep as transcripts are sequenced from the 3′ to 5′ end [27]. ONT has made error correction tools such as Nanopolish available to try and reduce the error rate observed with Nanopore sequencing [28]. In this study, variant calling was achieved using Nanopolish but we still note a significantly lower genome quality obtained from the GridION than the MiSeq. These low-quality genomes cannot be used to confidently acquire information on the infecting viral strain and are generally removed through a series of quality control checks [29]. Although more consensus genomes can be produced using the GridION than the MiSeq, the low-quality genomes which are removed would eliminate the advantage of having a large number of consensus genomes produced. It should be noted that the quality and coverage of consensus genomes for the ONT GridION can be increased by pooling lower samples as the number of reads and data produced will be shared across a smaller group.

Although Bull et al. 2020 shows that Nanopore sequencing was able to produce consensus genomes that were high quality, the SARS-CoV-2 viral variants that were available for analysis may not have been as diverse as the variants analysed in this study. This may have been due to the number of samples that were used for the study and the diversity of the samples as was as 157 samples were used in the study all of which came from Wales and Metropolitan Sydney. Furthermore, Samples were collected between March and April 2020 which may suggest that the viral variants in circulation were not as diverse as analysing samples from different African regions within a 1 year time frame as seen in this study.

Higher genomic coverage for the Illumina MiSeq has been associated with lower Ct scores [30]. Ct score is a value that refers to the number of cycles required to amplify viral RNA to a detectable level. There is therefore an inverse relationship between Ct score and viral load [31]. In this investigation, we also noted an inverse relationship between Ct score and genome coverage for both GridION and MiSeq sequencing. There is, however, a significantly stronger negative correlation seen with the GridION than the MiSeq, which may imply that the MiSeq’s sequencing capabilities are less affected by sample Ct score and as a result, can be used for sequencing of samples within the early stages of infection when viral load is still low. This was, however, limited by not having the same runs to compare between the GridION and the MiSeq. Further analysis is required as the number of samples analyzed for each run was low and inconsistent due to the availability of Ct scores received with sample metadata. Additional analyses should be conducted to understand characteristics such as coverage bias, sequence biases, and reproducibility for the GridION sequencing platform [26]. Sample quality may also have an effect on sequencing and thus it is very important to maintain a cold chain during storage of swabs and RNA.

Identifying mutations involves aligning a consensus genome to a reference genome and identifying changes within the consensus genome. This is important, as it allows us to identify gene variants that may play a major role in the diagnosis of diseases [32]. It has been shown that long-read sequencing platforms have a high error rate, which is mostly indels that are assumed to be randomly distributed within each read [33, 34]. Prediction and interpretation of protein sequences may, therefore, be critically affected due to frameshifts and premature stop codons that may be introduced by the indels [35].

There was a significantly greater number of mutations detected by the MiSeq than the GridION for identical samples sequenced on both platforms. Although Nanopore platforms have been shown to make a large number of indel errors, in this study the MiSeq had a significantly higher number of insertions than the GridION. Paired-end sequencing, utilized by Illumina MiSeq, produces twice the number of reads, for the same sample and library preparation efforts, as single-end sequencing. This allows for a more accurate read alignment and detection of indel variants [36]. Short read lengths have been shown to hinder the assignment of reads to parts of the genome that are complex, phasing of variants, resolving regions that are repeated, and the introduction of gaps and ambiguous regions in de novo assemblies. Longer reads can be used for sequencing of extended repetitive regions, allowing for the identification of mutations that are generally associated with disease [37]. The higher number of indels noted with GridION sequencing highlights that genomic surveillance using Nanopore sequencing should be conducted cautiously as incorrect information on a viral strain can be obtained.

The rapid increase in COVID-19 cases has been linked to different SARS-CoV-2 viral lineages [38]. Viral lineages are separated based on the number and type of mutations they contain that differ from the parent strain [39]. From the 93 consensus genomes analyzed from both platforms, 27 genomes were classified within different clades. These genomes had unique mutations and the clade differences noted between the two platforms were 20A – 20C and 20C – 20H(Beta, V2). As the number of indels and substitutions produced by the MiSeq and the GridION were significantly different, we can expect there to be differences in clade classifications as viral clades are subject to viral-defining mutations [20]. Table 2 shows that genomes from the GridION have lower coverages than genomes from the MiSeq. This may be one of the factors causing a difference in the clade assignment as errors arising from the amplification and sequencing process may result in incomplete genome coverage, which affects phylogenetic inference [40]. Rambaut et al., 2020 suggests that new lineages should only be proposed if the genome coverage exceeds 70% of the coding region. Degradation of RNA can result in the introduction of mutations, which may cause a variant change [41]. The GridION library for RUN116 was prepared simultaneously with that of the MiSeq and the amount of RNA used is also lower. Therefore, we can eliminate the possibility of RNA degradation and RNA input amount as factors that may have caused a difference in the variants called by each instrument. Lineages identified by the GridION need to be further analyzed to determine whether the mutations are valid or are a result of sequencing errors. Accurate identification of lineages can assist in identifying transmission chains and allow for the development of diagnostic methods and treatments [38].

## Conclusions

The results of this study show that the ONT GridION is less ideal for SARS-CoV-2 genomic surveillance than the Illumina MiSeq but can be used to produce consensus genomes from samples of high quality and low CT scores. Healthcare facilities can, however, use ONT sequencing platforms to rapidly diagnose patients as the GridION can sequence up to 480 samples every 21 h. This may allow for the identification and isolation of isolate infected individuals, thus aiding in stopping the spread of the disease.

## Methods

### Study population

The study population consisted of positive COVID-19 male and female patients whose nasopharyngeal swabs were sent from routine PCR diagnostic services for genomic surveillance to the Kwazulu-Natal Research Innovation and Sequencing Platform (KRISP). A total of 2608 COVID-19 positive nasopharyngeal swabs were used for sequencing from 28 different runs split evenly between the GridION and MiSeq. Samples were randomized and were from South Africa, Angola, Malawi, Mozambique, and Zimbabwe.

### Real-time PCR assays

Sample Ct scores were present in the metadata files accompanying samples brought in for sequencing. There were three RT-PCR assays used for these samples. Namely; Seegene-AllplexTM 2019-nCoV Assay, Roche-Cobas® SARS-CoV-2 Qualitative assay, and Thermofisher-TaqPath™ COVID 19 CE IVD RT PCR Kit.

### Total nucleic acid extraction

RNA was extracted using the NA/gDNA kit on the automated Chemagic 360 system (Perkin Elmer) as per the manufacturer’s instructions. Briefly, samples were lysed using lysis buffer and proteinase K, followed by binding to silica magnetic beads. The beads were then washed to remove unbound samples, and the RNA was eluted. Extracted RNA was stored at − 80 °C before use.

### Tiling PCR

Complementary DNA synthesis was performed using SuperScript IV reverse transcriptase (Life Technologies) in combination with random hexamer primers. This was then followed by gene-specific multiplex PCR using the ARTIC protocol [42]. Primers were designed on a primal scheme (http://primal.zebraproject.org/) to cover the SARS-CoV-2 whole genome. Primers generated were 400 base pair (bp) amplicons, with an overlap of 70 bp to cover the 30 kilobases (kb) SARS-CoV-2 genome. Purification of PCR products was performed using AmpureXP purification beads in a 1:1 ratio (Beckman Coulter, High Wycombe, UK) and quantification was performed using the Qubit double-strand DNA (dsDNA) High Sensitivity Assay Kit on a Qubit 4.0 instrument (Life Technologies).

### Illumina MiSeq library preparation and sequencing

Sequencing libraries were generated using the amplicons generated by tiling PCR as described above. Indexed paired-end libraries were prepared using the Nextera DNA Flex Library Prep Kits (Illumina) as per the manufacturer’s instructions. Briefly, amplicons were tagmented to allow for unfragmented DNA to be cleaved and tagged. Each sample was barcoded with a unique barcode using the Nextera CD Indexes (Illumina) to enable downstream pooling of all libraries. Libraries were purified and normalized to 4 nM prior to pooling. The pooled library was denatured using 0.2 N sodium acetate and then diluted to a final concentration of 8 pM. The library was spiked with 1% PhiX Control v3 (adapter-ligated library used as a control), and the libraries were sequenced using a 500-cycle v2 MiSeq Reagent Kit on the Illumina MiSeq instrument (Illumina, San Diego, CA, USA). The full details of the amplification and sequencing have been previously published [30]. Fastq files produced from Illumina MiSeq were assembled using Genome Detective (https://www.genomedetective.com/) and the coronavirus typing tool [43]. Genome detective is a web-based application that is user-friendly and is used for the assembly of known viral genomes from NGS datasets [43]. Fastq files are uploaded to the application and read quality is visualized using FastQC. Low-quality reads are then filtered and the adapters trimmed with Trimmomatic [44]. DIAMOND, a protein-based alignment method, is used to identify candidate viral reads [45]. The Swissprot UniRef90 protein database viral subset is used to improve speed and sensitivity [43]. Short reads are sorted and placed into groups and metagenomic de novo assembly is performed on each group using SPAdes for single-ended reads or metaSPAdes for paired-end reads [46]. Each group is then identified using the taxonomy ID of the lowest common ancestor of the hits identified by DIAMOND [45]. Blastx and Blastn are used to search for candidate reference sequences against the NCBI RefSeq virus database. The results for all detected contigs are combined by the Advanced Genome Aligner and a score is calculated by Genome Detective at the amino acid and nucleotide level. The five best scoring references for each config are then used for the alignment [43].

### ONT GridION library preparation and sequencing

Amplicons generated using the tiling PCR were prepared for nanopore sequencing using the ONT Native Barcoding Expansion Kits as per the manufacturer’s guidelines. Libraries were multiplexed on FLO-MIN106 flowcells and run on the GridION X5. Furthermore, a no-template control from the PCR amplification step was added to each plate before running. Sequencing performance was monitored, in real-time, using the MinKNOW software app. Sequencing was terminated after 21 h and the resulting reads were base-called using Guppy (4.0.14) and aligned to the Wuhan-Hu-1 reference genome (MN908947.3) using minimap2 (2.17-r941). Primer sequences were trimmed from the termini of read alignments and sequencing depth was capped at a maximum of 400-fold coverage using the ARTIC tool align_trim. Variant candidates were identified using Nanopolish [47].

### Sequence analysis

Consensus genomes produced by both platforms were uploaded to Nextclade Online Tool v1.4.2 (2021-10-26) (https://clades.nextstrain.org/) for genome clade assignments, mutation calling, quality checks, and to determine the genome position on the SARS-CoV-2 phylogenetic tree. Nextclade is built on Nextalign and consists of three tools; Nextclade Web, Nextclade CLI, and Nextalign CLI, which all share the common C++ library of algorithms. Nextclade starts by performing a pairwise alignment of the query sequence to a reference sequence using Nextalign that uses a banded local alignment algorithm with affine gap-cost that are determined through seed matching. Alignment is only performed on sequences longer than 100 nucleotides by default, but this can be changed, as alignment of shorter sequences may be unreliable. Mutation calling is achieved by comparing the aligned nucleotide sequences, one at a time, with the reference nucleotide sequence. Depending on their nature, they are reported differently. The number of missing, and ambiguous bases are also reported. Nextclade places each query sequence on the reference phylogenetic tree by comparing the mutations on the query sequence with the mutations of every node and tip in the reference tree, and finding the node which has the most similar set of mutations. Clade assignment is achieved by placing sequences on a phylogenetic tree annotated with clade definitions [48]. A Maximum-likelihood (ML) tree was constructed using IQ-TREE and was visualized using FigTree v1.4.4 (https://github.com/rambaut/figtree/releases) [49]. Data visualization and statistical analysis were performed using ggplot2 v3.3.1 package and R v.4.1.1.

### Statistical considerations

The non-parametric nature of the data influenced the use of a Wilcoxon test to compare the number of consensus genomes produced by the GridION and the MiSeq classified within each category of the online Nextclade sequence analysis tool. The Wilcoxon test was also used to compare the difference in genomic coverage, number, and type of mutations detected between the GridION and the MiSeq. Statistical correlations were performed between Ct score and genome coverage and Ct score and the number of reads for both platforms.

### Ethics

The University of KwaZulu-Natal Biomedical Research Ethics Committee waived the requirement for informed consent and approved the study (protocol reference no. BREC/00001195/2020; project title: COVID-19 transmission and natural history in KwaZulu-Natal, South Africa: epidemiological investigation to guide prevention and clinical care). All methods were performed in accordance with the relevant guidelines and regulations. We also used de-identified remnant nasopharyngeal and oropharyngeal swab samples from patients testing positive for SARS-CoV-2 by RT–qPCR from public health laboratories in South Africa. Informed consent for study participation was not applicable for this study because de-identified (anonymous) remnant samples, which would have been otherwise discarded, were used.

## Data Availability

The datasets generated and analysed during the current study are available in the SRA (https://www.ncbi.nlm.nih.gov/sra) and GISAID (https://www.gisaid.org/) data repositories. SRR14712306; SRR14712358; SRR14712352; SRR14712349; SRR14712347; SRR14712326; SRR14712370; SRR14712367; SRR14712351; SRR14712334; SRR14712322; SRR14712320; SRR14712319; SRR14712311; SRR14712366; SRR14712359; SRR14712355; SRR14712353; SRR14712337; SRR14712329; SRR14712325; SRR14712323; SRR14712321; SRR14712318; SRR14712315; SRR14712314; SRR14712308; SRR14712290; SRR14712361; SRR14712339; SRR14712365; SRR14712357; SRR14712336; SRR14712333; SRR14712312; SRR14712310; SRR14712307; SRR14712286; SRR14712283; SRR14712328; SRR14712301; SRR14712299; SRR14712293; SRR14712289; SRR14712300; SRR14712363; SRR14712360; SRR14712338; SRR14712331; SRR14712295; SRR14712284; SRR14712362; SRR14712354; SRR14712340; SRR14712294; SRR14712372; SRR14712317; SRR14712342; SRR14712335; SRR14712332; SRR14712309; SRR14712303; SRR14712302; SRR14712298; SRR14712297; SRR14712296; SRR14712292; SRR14712291; SRR14712285; SRR14712304; SRR14712373; SRR14712368; SRR14712364; SRR14712350; SRR14712356; SRR14712287; SRR14712313; SRR14712345; SRR14712346; SRR14712344; SRR14712369; SRR14712343; SRR14712305; SRR14712371; SRR14712288; SRR14712330; SRR14712341; SRR14712327; SRR14712348; SRR14712316; SRR14712324; SRR14711778; SRR14711777; SRR14711776; SRR14711775; SRR14711774; SRR14711773; SRR14711598; SRR14711599; SRR14711600; SRR14711771; SRR14711770; SRR14711769; SRR14711601; SRR14711602; SRR14711603; SRR14711604; SRR14711605; SRR14711606; SRR14711607; SRR14711766; SRR14711765; SRR14711608; SRR14711609; SRR14711610; SRR14711611; SRR14711612; SRR14711613; SRR14711614; SRR14711615; SRR14711617; SRR14711618; SRR14711619; SRR14711620; SRR14711645; SRR14711646; SRR14711647; SRR14711648; SRR14711649; SRR14711650; SRR14711652; SRR14711653; SRR14711654; SRR14711655; SRR14711656; SRR14711657; SRR14711658; SRR14711659; SRR14711660; SRR14711661; SRR14711763; SRR14711762; SRR14711761; SRR14711760; SRR14711759; SRR14711758; SRR14711757; SRR14711756; SRR14711755; SRR14711754; SRR14711752; SRR14711751; SRR14711750; SRR14711749; SRR14711748; SRR14711747; SRR14711746; SRR14711745; SRR14711744; SRR14711743; SRR14711725; SRR14711724; SRR14711723; SRR14711722; SRR14711721; SRR14711720; SRR14711719; SRR14711662; SRR14711663; SRR14711664; SRR14711717; SRR14711716; SRR14711715; SRR14711665; SRR14711666; SRR14711667; SRR14711668; SRR14711669; SRR14711670; SRR14711671; SRR14711713; SRR14711712; SRR14711711; SRR14711672; SRR14711673; SRR14711933; SRR14711969; SRR14711945; SRR14711907; SRR14711908; SRR14711911; SRR14711912; SRR14711919; SRR14711921; SRR14711925; SRR14711957; SRR14711910; SRR14711913; SRR14711916; SRR14711924; SRR14711930; SRR14711931; SRR14711901; SRR14711902; SRR14711966; SRR14711964; SRR14711956; SRR14711905; SRR14711909; SRR14711918; SRR14711968; SRR14711927; SRR14711936; SRR14711941; SRR14711949; SRR14711952; SRR14711903; SRR14711961; SRR14712046; SRR14711904; SRR14711929; SRR14711906; SRR14711914; SRR14711917; SRR14711920; SRR14711932; SRR14711943; SRR14711944; SRR14712048; SRR14712045; SRR14711975; SRR14711915; SRR14711974; SRR14711951; SRR14711963; SRR14711955; SRR14711922; SRR14712047; SRR14711940; SRR14711935; SRR14711938; SRR14711942; SRR14711946; SRR14711972; SRR14711967; SRR14711948; SRR14711971; SRR14711934; SRR14711928; SRR14711970; SRR14711939; SRR14711962; SRR14711965; SRR14711937; SRR14711958; SRR14711950; SRR14711959; SRR14711973; SRR14711926; SRR14711900; SRR14711954; SRR14711947; SRR14711953; SRR14711923; SRR14711960; SRR14709591; SRR14709524; SRR14709536; SRR14709545; SRR14709588; SRR14709552; SRR14709589; SRR14709549; SRR14709546; SRR14709539; SRR14709532; SRR14709528; SRR14709580; SRR14709570; SRR14709569; SRR14709566; SRR14709558; SRR14709541; SRR14709530; SRR14709553; SRR14709550; SRR14709551; SRR14709548; SRR14709547; SRR14709543; SRR14709542; SRR14709538; SRR14709537; SRR14709584; SRR14709579; SRR14709573; SRR14709572; SRR14709556; SRR14709590; SRR14709585; SRR14709574; SRR14709529; SRR14709527; SRR14709587; SRR14709575; SRR14709568; SRR14709567; SRR14709565; SRR14709564; SRR14709563; SRR14709544; SRR14709526; SRR14709578; SRR14709560; SRR14709555; SRR14709533; SRR14709525; SRR14709535; SRR14709586; SRR14709540; SRR14709531; SRR14709523; SRR14709561; SRR14709562; SRR14709554; SRR14709582; SRR14709534; SRR14709571; SRR14709583; SRR14709557; SRR14709559; SRR14709576; SRR14709577; SRR14709581; SRR14707695; SRR14707647; SRR14707690; SRR14707691; SRR14707710; SRR14707630; SRR14707633; SRR14707644; SRR14707654; SRR14707660; SRR14707668; SRR14707674; SRR14707675; SRR14707676; SRR14707680; SRR14707688; SRR14707631; SRR14707632; SRR14707645; SRR14707656; SRR14707657; SRR14707664; SRR14707666; SRR14707677; SRR14707683; SRR14707692; SRR14707693; SRR14707696; SRR14707699; SRR14707701; SRR14707709; SRR14707712; SRR14707713; SRR14707718; SRR14707648; SRR14707655; SRR14707658; SRR14707663; SRR14707681; SRR14707686; SRR14707687; SRR14707698; SRR14707714; SRR14707715; SRR14707716; SRR14707636; SRR14707646; SRR14707659; SRR14707667; SRR14707670; SRR14707694; SRR14707650; SRR14707700; SRR14707702; SRR14707671; SRR14707684; SRR14707661; SRR14707665; SRR14707679; SRR14707635; SRR14707637; SRR14707669; SRR14707640; SRR14707643; SRR14707711; SRR14707689; SRR14707634; SRR14707641; SRR14707653; SRR14707678; SRR14707697; SRR14707651; SRR14707706; SRR14707673; SRR14707717; SRR14707649; SRR14707652; SRR14707682; SRR14707662; SRR14707672; SRR14707705; SRR14707703; SRR14707704; SRR14707719; SRR14707708; SRR14707628; SRR14707638; SRR14707639; SRR14707685; SRR14707629; SRR14707642; SRR14707707; SRR14995823; SRR14995837; SRR14995845; SRR14995848; SRR14995857; SRR14995866; SRR14935762; SRR14935764; SRR14995873; SRR14995824; SRR14995838; SRR14995844; SRR14935757; SRR14995826; SRR14995828; SRR14935759; SRR14995831; SRR14995847; SRR14995906; SRR14995863; SRR14995818; SRR14995853; SRR14995895; SRR14995810; SRR14995799; SRR14995830; SRR14995864; SRR14935760; SRR14995822; SRR14995834; SRR14935771; SRR14935766; SRR14995858; SRR14995867; SRR14995884; SRR14935761; SRR14995839; SRR14995868; SRR14935767; SRR14995833; SRR14995841; SRR14995855; SRR14995843; SRR14995849; SRR14995852; SRR14995842; SRR14995859; SRR14995850; SRR14995854; SRR14995846; SRR14935758; SRR14995861; SRR14935773; SRR14935772; SRR14935770; SRR14995836; SRR14935768; SRR14995856; SRR14995835; SRR14935763; SRR14935754; SRR14935753; SRR14995832; SRR14995865; SRR14995869; SRR14995870; SRR14995871; SRR14995872; SRR14995874; SRR14995875; SRR14995876; SRR14995877; SRR14995878; SRR14995879; SRR14995880; SRR14995881; SRR14995882; SRR14995883; SRR14995885; SRR14995886; SRR14995887; SRR14995888; SRR14995889; SRR14995890; SRR14995891; SRR14995892; SRR14995893; SRR14995894; SRR14995896; SRR14995897; SRR14995898; SRR14995899; SRR14995900; SRR14995901; SRR14995902; SRR14995903; SRR14995904; SRR14995905; SRR14995907; SRR14995908; SRR14995909; SRR14995910; SRR14995911; SRR14995912; SRR14995913; SRR14995914; SRR14995915; SRR14995916; SRR14995800; SRR14995801; SRR14995802; SRR14995803; SRR14995804; SRR14995805; SRR14995806; SRR14995807; SRR14995808; SRR14995809; SRR14995811; SRR14995812; SRR14995813; SRR14995814; SRR14995815; SRR14995816; SRR14995924; SRR14995923; SRR14995922; SRR14995819; SRR14995820; SRR14995821; SRR14995921; SRR14995920; SRR14995919; SRR14995918; SRR14995917; SRR15005052; SRR15003744; SRR15003770; SRR15003768; SRR15003759; SRR15003811; SRR15003801; SRR15003734; SRR15003803; SRR15003762; SRR15003779; SRR15003752; SRR15003782; SRR15003717; SRR15003815; SRR15003792; SRR15003772; SRR15003767; SRR15003718; SRR15003748; SRR15003766; SRR15003738; SRR15003739; SRR15003771; SRR15003769; SRR15003755; SRR15003760; SRR15003780; SRR15003763; SRR15003756; SRR15003716; SRR15003753; SRR15003751; SRR15003794; SRR15003722; SRR15003807; SRR15003729; SRR15003798; SRR15003793; SRR15003747; SRR15003808; SRR15003737; SRR15003735; SRR15003730; SRR15003806; SRR15003791; SRR15003749; SRR15003731; SRR15003797; SRR15003777; SRR15003813; SRR15003800; SRR15003785; SRR15003781; SRR15003764; SRR15003741; SRR15003727; SRR15003724; SRR15003719; SRR15003814; SRR15003784; SRR15003778; SRR15003728; SRR15003720; SRR15003789; SRR15003761; SRR15003757; SRR15003745; SRR15003715; SRR15003796; SRR15003774; SRR15003736; SRR15003726; SRR15003775; SRR15003723; SRR15003802; SRR15003714; SRR15003786; SRR15003733; SRR15003725; SRR15003809; SRR15003742; SRR15003740; SRR15003812; SRR15003795; SRR15003773; SRR15003750; SRR15003746; SRR15003804; SRR15003783; SRR15003805; SRR15003790; SRR15003758; SRR14189637; SRR14189636; SRR14189625; SRR14189614; SRR14189603; SRR14189592; SRR14189581; SRR14189570; SRR14189559; SRR14189548; SRR14189635; SRR14189634; SRR14189633; SRR14189632; SRR14189631; SRR14189630; SRR14189629; SRR14189628; SRR14189627; SRR14189626; SRR14189624; SRR14189623; SRR14189622; SRR14189621; SRR14189620; SRR14189619; SRR14189618; SRR14189617; SRR14189616; SRR14189615; SRR14189613; SRR14189612; SRR14189611; SRR14189610; SRR14189609; SRR14189608; SRR14189607; SRR14189606; SRR14189605; SRR14189604; SRR14189602; SRR14189601; SRR14189600; SRR14189599; SRR14189598; SRR14189597; SRR14189596; SRR14189595; SRR14189594; SRR14189593; SRR14189591; SRR14189590; SRR14189589; SRR14189588; SRR14189587; SRR14189586; SRR14189585; SRR14189584; SRR14189583; SRR14189582; SRR14189580; SRR14189579; SRR14189578; SRR14189577; SRR14189576; SRR14189575; SRR14189574; SRR14189573; SRR14189572; SRR14189571; SRR14189569; SRR14189568; SRR14189567; SRR14189566; SRR14189565; SRR14189564; SRR14189563; SRR14189562; SRR14189561; SRR14189560; SRR14189558; SRR14189557; SRR14189556; SRR14189555; SRR14189554; SRR14189553; SRR14189552; SRR14189551; SRR14189550; SRR14189549; SRR14189547; SRR14189546; SRR14189545; SRR14189544; SRR14272078; SRR14272077; SRR14272066; SRR14272055; SRR14272044; SRR14272033; SRR14272022; SRR14272011; SRR14272000; SRR14271989; SRR14272076; SRR14272075; SRR14272074; SRR14272073; SRR14272072; SRR14272071; SRR14272070; SRR14272069; SRR14272068; SRR14272067; SRR14272065; SRR14272064; SRR14272063; SRR14272062; SRR14272061; SRR14272060; SRR14272059; SRR14272058; SRR14272057; SRR14272056; SRR14272054; SRR14272053; SRR14272052; SRR14272051; SRR14272050; SRR14272049; SRR14272048; SRR14272047; SRR14272046; SRR14272045; SRR14272043; SRR14272042; SRR14272041; SRR14272040; SRR14272039; SRR14272038; SRR14272037; SRR14272036; SRR14272035; SRR14272034; SRR14272032; SRR14272031; SRR14272030; SRR14272029; SRR14272028; SRR14272027; SRR14272026; SRR14272025; SRR14272024; SRR14272023; SRR14272021; SRR14272020; SRR14272019; SRR14272018; SRR14272017; SRR14272016; SRR14272015; SRR14272014; SRR14272013; SRR14272012; SRR14272010; SRR14272009; SRR14272008; SRR14272007; SRR14272006; SRR14272005; SRR14272004; SRR14272003; SRR14272002; SRR14272001; SRR14271999; SRR14271998; SRR14271997; SRR14271996; SRR14271995; SRR14271994; SRR14271993; SRR14271992; SRR14271991; SRR14271990; SRR14271988; SRR14271987; SRR14271986; SRR14271985; SRR14271984; SRR14271983; SRR14271972; SRR14271965; SRR14271964; SRR14271963; SRR14271962; SRR14271961; SRR14271960; SRR14271959; SRR14271982; SRR14271981; SRR14271980; SRR14271979; SRR14271978; SRR14271977; SRR14271976; SRR14271975; SRR14271974; SRR14271973; SRR14271971; SRR14271970; SRR14271969; SRR14271968; SRR14271967; SRR14271966; SRR14272146; SRR14272145; SRR14272134; SRR14272123; SRR14272112; SRR14272101; SRR14272090; SRR14272081; SRR14272080; SRR14272079; SRR14272144; SRR14272143; SRR14272142; SRR14272141; SRR14272140; SRR14272139; SRR14272138; SRR14272137; SRR14272136; SRR14272135; SRR14272133; SRR14272132; SRR14272131; SRR14272130; SRR14272129; SRR14272128; SRR14272127; SRR14272126; SRR14272125; SRR14272124; SRR14272122; SRR14272121; SRR14272120; SRR14272119; SRR14272118; SRR14272117; SRR14272116; SRR14272115; SRR14272114; SRR14272113; SRR14272111; SRR14272110; SRR14272109; SRR14272108; SRR14272107; SRR14272106; SRR14272105; SRR14272104; SRR14272103; SRR14272102; SRR14272100; SRR14272099; SRR14272098; SRR14272097; SRR14272096; SRR14272095; SRR14272094; SRR14272093; SRR14272092; SRR14272091; SRR14272089; SRR14272088; SRR14272087; SRR14272086; SRR14272085; SRR14272084; SRR14272083; SRR14272082; SRR14271958; SRR14271957; SRR14271946; SRR14271935; SRR14271924; SRR14271913; SRR14271902; SRR14271891; SRR14271880; SRR14271869; SRR14271956; SRR14271955; SRR14271954; SRR14271953; SRR14271952; SRR14271951; SRR14271950; SRR14271949; SRR14271948; SRR14271947; SRR14271945; SRR14271944; SRR14271943; SRR14271942; SRR14271941; SRR14271940; SRR14271939; SRR14271938; SRR14271937; SRR14271936; SRR14271934; SRR14271933; SRR14271932; SRR14271931; SRR14271930; SRR14271929; SRR14271928; SRR14271927; SRR14271926; SRR14271925; SRR14271923; SRR14271922; SRR14271921; SRR14271920; SRR14271919; SRR14271918; SRR14271917; SRR14271916; SRR14271915; SRR14271914; SRR14271912; SRR14271911; SRR14271910; SRR14271909; SRR14271908; SRR14271907; SRR14271906; SRR14271905; SRR14271904; SRR14271903; SRR14271901; SRR14271900; SRR14271899; SRR14271898; SRR14271897; SRR14271896; SRR14271895; SRR14271894; SRR14271893; SRR14271892; SRR14271890; SRR14271889; SRR14271888; SRR14271887; SRR14271886; SRR14271885; SRR14271884; SRR14271883; SRR14271882; SRR14271881; SRR14271879; SRR14271878; SRR14271877; SRR14271876; SRR14271875; SRR14271874; SRR14271873; SRR14271872; SRR14271871; SRR14271870; SRR14271868; SRR14271867; SRR14271866; SRR14271865; SRR14663140; SRR14663139; SRR14663128; SRR14663122; SRR14663121; SRR14663120; SRR14663119; SRR14663118; SRR14663117; SRR14663116; SRR14663138; SRR14663137; SRR14663136; SRR14663135; SRR14663134; SRR14663133; SRR14663132; SRR14663131; SRR14663130; SRR14663129; SRR14663127; SRR14663126; SRR14663125; SRR14663124; SRR14663123; SRR14401102; SRR14401101; SRR14401099; SRR14401098; SRR14401097; SRR14401096; SRR14401095; SRR14401094; SRR14401093; SRR14401092; SRR14401091; SRR14401090; SRR14401064; SRR14401063; SRR14401062; SRR14401061; SRR14401060; SRR14401059; SRR14401058; SRR14401057; SRR14401056; SRR14401055; SRR14401052; SRR14401051; SRR14401050; SRR14401049; SRR14401048; SRR14401047; SRR14401046; SRR14401045; SRR14401044; SRR14401043; SRR14401041; SRR14401040; SRR14401039; SRR14401038; SRR14401013; SRR14401012; SRR14401011; SRR14401010; SRR14401009; SRR14401008; SRR14401006; SRR14401005; SRR14401004; SRR14401003; SRR14401002; SRR14401001; SRR14401000; SRR14400999; SRR14400998; SRR14400997; SRR14400995; SRR14400994; SRR14400993; SRR14400992; SRR14400991; SRR14400990; SRR14400989; SRR14400988; SRR14400987; SRR14400986; SRR14400984; SRR14400983; SRR14400982; SRR14400981; SRR14400980; SRR14400979; SRR14400978; SRR14455054; SRR14455046; SRR14455117; SRR14455055; SRR14455081; SRR14455037; SRR14455064; SRR14455122; SRR14455123; SRR14455057; SRR14455047; SRR14455103; SRR14455049; SRR14455126; SRR14455125; SRR14455070; SRR14455124; SRR14455051; SRR14455111; SRR14455056; SRR14455120; SRR14455119; SRR14455113; SRR14455112; SRR14455105; SRR14455068; SRR14455044; SRR14455098; SRR14455109; SRR14455102; SRR14455100; SRR14455066; SRR14455067; SRR14455065; SRR14455062; SRR14455059; SRR14455121; SRR14455050; SRR14455048; SRR14455118; SRR14455116; SRR14455115; SRR14455097; SRR14455063; SRR14455110; SRR14455108; SRR14455104; SRR14455069; SRR14455043; SRR14455042; SRR14455041; SRR14455106; SRR14455099; SRR14455061; SRR14455058; SRR14455053; SRR14455038; SRR14455107; SRR14455040; SRR14455101; SRR14455085; SRR14455086; SRR14455094; SRR14455093; SRR14455088; SRR14455079; SRR14455114; SRR14455096; SRR14455095; SRR14455091; SRR14455087; SRR14455084; SRR14455078; SRR14455076; SRR14455074; SRR14455090; SRR14455089; SRR14455083; SRR14455082; SRR14455077; SRR14455075; SRR14455072; SRR14455073; SRR14455045; SRR14455039; SRR14455036; SRR14455080; SRR14455060; SRR14455052; SRR14455092; SRR14455071; SRR14455035; SRR14584921; SRR14584941; SRR14584926; SRR14584913; SRR14584933; SRR14584940; SRR14584937; SRR14584928; SRR14584919; SRR14590511; SRR14590491; SRR14584943; SRR14584903; SRR14584927; SRR14584914; SRR14590517; SRR14584930; SRR14584912; SRR14590512; SRR14590510; SRR14590509; SRR14584936; SRR14584925; SRR14584934; SRR14584938; SRR14590506; SRR14590494; SRR14584935; SRR14584922; SRR14584944; SRR14584910; SRR14584901; SRR14584924; SRR14584923; SRR14584942; SRR14584929; SRR14584909; SRR14584907; SRR14590493; SRR14590513; SRR14590496; SRR14584902; SRR14584900; SRR14584911; SRR14590508; SRR14590497; SRR14590520; SRR14590515; SRR14590502; SRR14590501; SRR14584931; SRR14590500; SRR14590516; SRR14584916; SRR14590507; SRR14590522; SRR14584920; SRR14584917; SRR14590503; SRR14590498; SRR14584918; SRR14590523; SRR14590505; SRR14584908; SRR14584906; SRR14590495; SRR14590521; SRR14590514; SRR14590499; SRR14584904; SRR14590504; SRR14584915; SRR14584905; SRR14584939; SRR14590518; SRR14584932; SRR14590492; SRR14590519; SRR14411491; SRR14411436; SRR14411464; SRR14411382; SRR14411457; SRR14411459; SRR14411447; SRR14411415; SRR14411454; SRR14411433; SRR14411503; SRR14411393; SRR14411502; SRR14411469; SRR14411451; SRR14411444; SRR14411443; SRR14411432; SRR14411452; SRR14411435; SRR14411442; SRR14411440; SRR14411404; SRR14411446; SRR14411439; SRR14411504; SRR14411458; SRR14411456; SRR14411448; SRR14411465; SRR14411455; SRR14411461; SRR14411480; SRR14411462; SRR14411426; SRR14411441; SRR14411445; SRR14411460; SRR14411434; SRR14411449; SRR14411453; SRR14411437; SRR14411463; SRR14411438; SRR14411450; SRR14411491; SRR14411436; SRR14411464; SRR14411382; SRR14411457; SRR14411459; SRR14411447; SRR14411415; SRR14411454; SRR14411433; SRR14411503; SRR14411393; SRR14411502; SRR14411469; SRR14411451; SRR14411444; SRR14411443; SRR14411432; SRR14411452; SRR14411435; SRR14411442; SRR14411440; SRR14411404; SRR14411446; SRR14411439; SRR14411504; SRR14411458; SRR14411456; SRR14411448; SRR14411465; SRR14411455; SRR14411461; SRR14411480; SRR14411462; SRR14411426; SRR14411441; SRR14411445; SRR14411460; SRR14411434; SRR14411449; SRR14411453; SRR14411437; SRR14411463; SRR14411438; SRR14411450; SRR14411491; SRR14411436; SRR14411464; SRR14411382; SRR14411457; SRR14411459; SRR14411447; SRR14411415; SRR14411454; SRR14411433; SRR14411503; SRR14411393; SRR14411502; SRR14411469; SRR14411451; SRR14411444; SRR14411443; SRR14411432; SRR14411452; SRR14411435; SRR14411442; SRR14411440; SRR14411404; SRR14411446; SRR14411439; SRR14411504; SRR14411458; SRR14411456; SRR14411448; SRR14411465; SRR14411455; SRR14411461; SRR14411480; SRR14411462; SRR14411426; SRR14411441; SRR14411445; SRR14411460; SRR14411434; SRR14411449; SRR14411453; SRR14411437; SRR14411463; SRR14411438; SRR14411450; SRR14633034; SRR14633080; SRR14633066; SRR14633065; SRR14633063; SRR14633061; SRR14633056; SRR14633055; SRR14633043; SRR14633041; SRR14633037; SRR14633035; SRR14633029; SRR14633027; SRR14633019; SRR14633007; SRR14633031; SRR14633058; SRR14633052; SRR14633044; SRR14633038; SRR14633036; SRR14633033; SRR14633032; SRR14633086; SRR14633075; SRR14633048; SRR14633047; SRR14633009; SRR14633006; SRR14633085; SRR14633084; SRR14633074; SRR14633028; SRR14633014; SRR14633079; SRR14633077; SRR14633072; SRR14633076; SRR14633026; SRR14633012; SRR14633042; SRR14633022; SRR14633018; SRR14633015; SRR14633030; SRR14633064; SRR14633051; SRR14633083; SRR14633069; SRR14633045; SRR14633082; SRR14633073; SRR14633062; SRR14633024; SRR14633046; SRR14633040; SRR14633057; SRR14633054; SRR14633020; SRR14633070; SRR14633060; SRR14633081; SRR14633078; SRR14633067; SRR14633021; SRR14633008; SRR14633050; SRR14633068; SRR14633017; SRR14633010; SRR14633059; SRR14633049; SRR14633071; SRR14633016; SRR14633025; SRR14633039; SRR14633023; SRR14736721; SRR14711714; SRR14736753; SRR14736683; SRR14736707; SRR14736706; SRR14736705; SRR14711596; SRR14711573; SRR14711753; SRR14736704; SRR14711685; SRR14711623; SRR14711584; SRR14711616; SRR14736715; SRR14736773; SRR14711698; SRR14711768; SRR14711710; SRR14711837; SRR14711726; SRR14736744; SRR14736762; SRR14711675; SRR14736775; SRR14736761; SRR14711696; SRR14711767; SRR14736723; SRR14711780; SRR14736774; SRR14736763; SRR14711812; SRR14711737; SRR14736766; SRR14736702; SRR14736751; SRR14736772; SRR14736757; SRR14736756; SRR14711699; SRR14736714; SRR14711772; SRR14736771; SRR14736720; SRR14736760; SRR14736755; SRR14736710; SRR14736752; SRR14736699; SRR14736713; SRR14736712; SRR14736711; SRR14736759; SRR14736694; SRR14736770; SRR14736758; SRR14736719; SRR14736769; SRR14736718; SRR14736708; SRR14736767; SRR14711651; SRR14736701; SRR14736700; SRR14736776; SRR14736765; SRR14736754; SRR14736716; SRR14711826; SRR14736709; SRR14711674; SRR14736777; SRR14736717; SRR14736764; SRR14736768; SRR14736722; SRR14736703; SRR14711801; SRR14711718; SRR14711791; SRR14711697; SRR14711634; SRR14711764; SRR14736733; SRR14720376; SRR14720324; SRR14720337; SRR14711624; SRR14711578; SRR14711836; SRR14711833; SRR14720335; SRR14720362; SRR14720378; SRR14720336; SRR14720338; SRR14720339; SRR14711577; SRR14711840; SRR14711832; SRR14720373; SRR14720375; SRR14711589; SRR14711787; SRR14711782; SRR14720360; SRR14720322; SRR14711586; SRR14711579; SRR14711575; SRR14711845; SRR14711844; SRR14711842; SRR14711838; SRR14711781; SRR14711828; SRR14711793; SRR14711580; SRR14711835; SRR14711824; SRR14711843; SRR14720374; SRR14711821; SRR14711621; SRR14720383; SRR14720381; SRR14711786; SRR14720333; SRR14720365; SRR14711794; SRR14711790; SRR14711625; SRR14711834; SRR14711591; SRR14711581; SRR14711789; SRR14711839; SRR14711831; SRR14711792; SRR14720361; SRR14711841; SRR14711583; SRR14711574; SRR14711846; SRR14720323; SRR14711829; SRR14711827; SRR14711825; SRR14720379; SRR14720382; SRR14711784; SRR14736697; SRR14736696; SRR14711592; SRR14711783; SRR14711590; SRR14711588; SRR14736695; SRR14736698; SRR14720325; SRR14711785; SRR14711823; SRR14711795; SRR14711830; SRR14711788; SRR14711622; SRR14711779; SRR14711822; SRR14711820; SRR14720380; SRR14711847; SRR14711576; SRR14736693; SRR14711582; SRR14711585; SRR14711587; SRR14720377; SRR14697479; SRR14697559; SRR14697548; SRR14697515; SRR14697513; SRR14697555; SRR14697540; SRR14697536; SRR14697525; SRR14697539; SRR14697529; SRR14697519; SRR14697485; SRR14697561; SRR14697494; SRR14697483; SRR14697554; SRR14697552; SRR14697532; SRR14697518; SRR14697509; SRR14697501; SRR14697499; SRR14697550; SRR14697510; SRR14697502; SRR14697496; SRR14697538; SRR14697505; SRR14697498; SRR14697490; SRR14697489; SRR14697551; SRR14697546; SRR14697517; SRR14697506; SRR14697482; SRR14697523; SRR14697484; SRR14697553; SRR14697547; SRR14697512; SRR14697516; SRR14697544; SRR14697514; SRR14697503; SRR14697507; SRR14697522; SRR14697527; SRR14697493; SRR14697549; SRR14697542; SRR14697543; SRR14697530; SRR14697541; SRR14697524; SRR14697520; SRR14697545; SRR14697521; SRR14697504; SRR14697491; SRR14697560; SRR14697508; SRR14697486; SRR14697533; SRR14697492; SRR14697480; SRR14697481; SRR14697537; SRR14697487; SRR14697556; SRR14697511; SRR14697488; SRR14697531; SRR14697535; SRR14697557; SRR14697497; SRR14697534; SRR14697500; SRR14697526; SRR14697558; SRR14697528; SRR14697495; SRR14697186; SRR14697153; SRR14697145; SRR14697148; SRR14697155; SRR14697128; SRR14697195; SRR14697167; SRR14697144; SRR14697143; SRR14697138; SRR14697191; SRR14697184; SRR14697170; SRR14697137; SRR14697168; SRR14697130; SRR14697127; SRR14697123; SRR14697122; SRR14697120; SRR14697197; SRR14697161; SRR14697157; SRR14697119; SRR14697192; SRR14697193; SRR14697154; SRR14697141; SRR14697147; SRR14697190; SRR14697185; SRR14697182; SRR14697179; SRR14697165; SRR14697150; SRR14697149; SRR14697172; SRR14697132; SRR14697199; SRR14697194; SRR14697189; SRR14697177; SRR14697204; SRR14697146; SRR14697169; SRR14697160; SRR14697156; SRR14697164; SRR14697133; SRR14697125; SRR14697136; SRR14697142; SRR14697174; SRR14697163; SRR14697152; SRR14697121; SRR14697140; SRR14697135; SRR14697124; SRR14697180; SRR14697178; SRR14697198; SRR14697175; SRR14697139; SRR14697131; SRR14697187; SRR14697134; SRR14697126; SRR14697181; SRR14697196; SRR14697162; SRR14697203; SRR14697176; SRR14697201; SRR14697171; SRR14697183; SRR14697166; SRR14697173; SRR14697188; SRR14697159; SRR14697200; SRR14697202; SRR14697158; SRR14697151; SRR14697118; SRR14707795; SRR14707742; SRR14707772; SRR14707791; SRR14707725; SRR14707805; SRR14707798; SRR14707723; SRR14707774; SRR14707758; SRR14707796; SRR14707752; SRR14707754; SRR14707756; SRR14707806; SRR14707780; SRR14707749; SRR14707802; SRR14707733; SRR14707745; SRR14707801; SRR14707729; SRR14707803; SRR14707784; SRR14707734; SRR14707739; SRR14707727; SRR14707732; SRR14707740; SRR14707724; SRR14707810; SRR14707808; SRR14707720; SRR14707722; SRR14707737; SRR14707766; SRR14707792; SRR14707738; SRR14707751; SRR14707744; SRR14707771; SRR14707809; SRR14707743; SRR14707762; SRR14707804; SRR14707787; SRR14707726; SRR14707765; SRR14707789; SRR14707741; SRR14707731; SRR14707799; SRR14707800; SRR14707757; SRR14707794; SRR14707746; SRR14707773; SRR14707770; SRR14707788; SRR14707779; SRR14707778; SRR14707769; SRR14707797; SRR14707785; SRR14707747; SRR14707768; SRR14707763; SRR14707777; SRR14707783; SRR14707735; SRR14707775; SRR14707761; SRR14707748; SRR14707753; SRR14707760; SRR14707807; SRR14707750; SRR14707793; SRR14707759; SRR14707736; SRR14707786; SRR14707767; SRR14707776; SRR14707781; SRR14707721; SRR14707782; SRR14707790; SRR14707730; SRR14707728; SRR14720357; SRR14736689; SRR14736688; SRR14736738; SRR14736727; SRR14736675; SRR14720372; SRR14736690; SRR14736687; SRR14736740; SRR14720368; SRR14736680; SRR14736748; SRR14736743; SRR14736741; SRR14736735; SRR14720348; SRR14736679; SRR14736747; SRR14736729; SRR14736692; SRR14720359; SRR14736684; SRR14736734; SRR14736745; SRR14736676; SRR14720363; SRR14736746; SRR14736682; SRR14736732; SRR14720364; SRR14720370; SRR14736750; SRR14720371; SRR14720331; SRR14720358; SRR14720344; SRR14720329; SRR14720330; SRR14720351; SRR14736724; SRR14720342; SRR14720334; SRR14720327; SRR14736691; SRR14720352; SRR14720346; SRR14736749; SRR14736742; SRR14720347; SRR14720355; SRR14736739; SRR14736728; SRR14736726; SRR14736686; SRR14736685; SRR14720343; SRR14720349; SRR14720345; SRR14720354; SRR14720366; SRR14736737; SRR14720341; SRR14720369; SRR14720326; SRR14720350; SRR14736678; SRR14720340; SRR14720356; SRR14736677; SRR14736681; SRR14736730; SRR14720367; SRR14736725; SRR14736736; SRR14720328; SRR14720332; SRR14720353; SRR14736731; SRR14757965; SRR14757940; SRR14757843; SRR14757936; SRR14757966; SRR14757917; SRR14757910; SRR14757847; SRR14757927; SRR14757926; SRR14757914; SRR14757920; SRR14757821; SRR14757962; SRR14757953; SRR14757946; SRR14757929; SRR14757830; SRR14757913; SRR14757943; SRR14757922; SRR14757915; SRR14757829; SRR14757834; SRR14757826; SRR14757916; SRR14757911; SRR14757825; SRR14757937; SRR14757935; SRR14757924; SRR14757919; SRR14757909; SRR14757959; SRR14757931; SRR14757844; SRR14757939; SRR14757925; SRR14757921; SRR14757918; SRR14757822; SRR14757819; SRR14757817; SRR14757961; SRR14757951; SRR14757947; SRR14757837; SRR14757957; SRR14757840; SRR14757950; SRR14757949; SRR14757944; SRR14757271; SRR14757832; SRR14757831; SRR14757272; SRR14757270; SRR14757846; SRR14757842; SRR14757828; SRR14757942; SRR14757273; SRR14757839; SRR14757835; SRR14757928; SRR14757941; SRR14757960; SRR14757938; SRR14757933; SRR14757958; SRR14757954; SRR14757836; SRR14757818; SRR14757952; SRR14757948; SRR14757932; SRR14757930; SRR14757833; SRR14757820; SRR14757955; SRR14757824; SRR14757845; SRR14757963; SRR14757841.

## Abbreviations

SARS-CoV-2: Severe Acute Respiratory Syndrome Corona Virus 2
WHO: World Health Organization
NGS: Next Generation Sequencing
GISAID: Global Initiative on Sharing All Influenza Data
ONT: Oxford Nanopore Technology
WGS: Whole Genome Sequencing
COVID-19: Corona Virus Disease 2019
KRISP: Kwazulu-Natal Research Innovation and Sequencing Platform
RNA: Ribonucleic Acid
DNA: Deoxyribonucleic Acid
PCR: Polymerase Chain Reaction
dsDNA: Double-Stranded Deoxyribonucleic Acid
ML: Maximum Likelihood
Ct: Cycle Threshold
QC: Quality Control
Ns: Non-significant

## References

[1] World Health O. Clinical management of severe acute respiratory infection when novel coronavirus (2019-nCoV) infection is suspected: interim guidance, 28 January 2020. Geneva: World Health Organization; 2020 2020. Contract No.: WHO/nCoV/Clinical/2020.3.

[2] Zhu N, Zhang D, Wang W, Li X, Yang B, Song J (2020). A novel coronavirus from patients with pneumonia in China, 2019. N Engl J Med.

[3] Esbin MN, Whitney ON, Chong S, Maurer A, Darzacq X, Tjian R (2020). Overcoming the bottleneck to widespread testing: a rapid review of nucleic acid testing approaches for COVID-19 detection. RNA.

[4] World Health O. WHO Director-General's opening remarks at the media briefing on COVID-19 - 11 March 2020 2020 [Available from: https://www.who.int/director-general/speeches/detail/who-director-general-s-opening-remarks-at-the-media-briefing-on-covid-19%2D%2D-11-march-2020.

[5] Amiel-Tison C (1978). Some aspects of prevention in perinatology (author's transl). J Gynecol Obstet Biol Reprod (Paris).

[6] Seth-Smith HMB, Bonfiglio F, Cuenod A, Reist J, Egli A, Wuthrich D (2019). Evaluation of rapid library preparation protocols for whole genome sequencing based outbreak investigation. Front Public Health.

[7] St Hilaire BG, Durand NC, Mitra N, Pulido SG, Mahajan R, Blackburn A, et al. A rapid, low cost, and highly sensitive SARS-CoV-2 diagnostic based on whole genome sequencing. bioRxiv. 2020:2020.04.25.061499. 10.1371/journal.pone.0294283 PMC10688730 38032990

[8] Liu L, Li Y, Li S, Hu N, He Y, Pong R (2012). Comparison of next-generation sequencing systems. J Biomed Biotechnol.

[9] GISAID. Pandemic coronavirus causing COVID-19 2022 [Available from: https://www.gisaid.org/.

[10] Chantal Babb de Villiers LB, Cook S, Janus J, Johnson E, Kroese M. Next generation sequencing for SARS-CoV-2. PHG Foundation. 2021.

[11] Resende PC, Motta FC, Roy S, Appolinario L, Fabri A, Xavier J, et al. SARS-CoV-2 genomes recovered by long amplicon tiling multiplex approach using nanopore sequencing and applicable to other sequencing platforms. bioRxiv. 2020:2020.04.30.069039.

[12] Gohl DM, Garbe J, Grady P, Daniel J, Watson RHB, Auch B (2020). A rapid, cost-effective tailed amplicon method for sequencing SARS-CoV-2. BMC Genomics.

[13] van Dijk EL, Jaszczyszyn Y, Naquin D, Thermes C (2018). The third revolution in sequencing technology. Trends Genet.

[14] Xu Y, Lewandowski K, Jeffery K, Downs LO, Foster D, Sanderson ND (2020). Nanopore metagenomic sequencing to investigate nosocomial transmission of human metapneumovirus from a unique genetic group among haematology patients in the United Kingdom. J Inf Secur.

[15] Schadt EE, Turner S, Kasarskis A (2010). A window into third-generation sequencing. Hum Mol Genet.

[16] Wang M, Fu A, Hu B, Tong Y, Liu R, Gu J, et al. Nanopore target sequencing for accurate and comprehensive detection of SARS-CoV-2 and other respiratory viruses. medRxiv. 2020:2020.03.04.20029538.

[17] Goodwin S, McPherson JD, McCombie WR (2016). Coming of age: ten years of next-generation sequencing technologies. Nat Rev Genet.

[18] Bull RA, Adikari TN, Ferguson JM, Hammond JM, Stevanovski I, Beukers AG (2020). Analytical validity of nanopore sequencing for rapid SARS-CoV-2 genome analysis. Nat Commun.

[19] Jayamohan H, Lambert CJ, Sant HJ, Jafek A, Patel D, Feng H (2021). SARS-CoV-2 pandemic: a review of molecular diagnostic tools including sample collection and commercial response with associated advantages and limitations. Anal Bioanal Chem.

[20] Tegally H, Wilkinson E, Lessells RJ, Giandhari J, Pillay S, Msomi N (2021). Sixteen novel lineages of SARS-CoV-2 in South Africa. Nat Med.

[21] James P, Stoddart D, Harrington ED, Beaulaurier J, Ly L, Reid SW, et al. LamPORE: rapid, accurate and highly scalable molecular screening for SARS-CoV-2 infection, based on nanopore sequencing. medRxiv. 2020:2020.08.07.20161737.

[22] Kono N, Arakawa K (2019). Nanopore sequencing: review of potential applications in functional genomics. Develop Growth Differ.

[23] Jain M, Koren S, Miga KH, Quick J, Rand AC, Sasani TA (2018). Nanopore sequencing and assembly of a human genome with ultra-long reads. Nat Biotechnol.

[24] Hourdel V, Kwasiborski A, Baliere C, Matheus S, Batejat CF, Manuguerra JC (2020). Rapid genomic characterization of SARS-CoV-2 by direct amplicon-based sequencing through comparison of MinION and Illumina iSeq100(TM) system. Front Microbiol.

[25] McNaughton AL, Roberts HE, Bonsall D, de Cesare M, Mokaya J, Lumley SF (2019). Illumina and Nanopore methods for whole genome sequencing of hepatitis B virus (HBV). Sci Rep.

[26] Amarasinghe SL, Su S, Dong X, Zappia L, Ritchie ME, Gouil Q (2020). Opportunities and challenges in long-read sequencing data analysis. Genome Biol.

[27] Sharon D, Tilgner H, Grubert F, Snyder M (2013). A single-molecule long-read survey of the human transcriptome. Nat Biotechnol.

[28] Loman NJ, Quick J, Simpson JT (2015). A complete bacterial genome assembled de novo using only nanopore sequencing data. Nat Methods.

[29] Gleizes A, Laubscher F, Guex N, Iseli C, Junier T, Cordey S (2020). Virosaurus a reference to explore and capture virus genetic diversity. Viruses.

[30] Pillay S, Giandhari J, Tegally H, Wilkinson E, Chimukangara B, Lessells R, et al. Whole genome sequencing of SARS-CoV-2: adapting Illumina protocols for Quick and accurate outbreak investigation during a pandemic. Genes (Basel). 2020;11(8). 10.3390/genes11080949 PMC7464704 32824573

[31] Tom MR, Mina MJ (2020). To interpret the SARS-CoV-2 test, consider the cycle threshold value. Clin Infect Dis.

[32] Wei X, Ju X, Yi X, Zhu Q, Qu N, Liu T (2011). Identification of sequence variants in genetic disease-causing genes using targeted next-generation sequencing. PLoS One.

[33] Ardui S, Ameur A, Vermeesch JR, Hestand MS (2018). Single molecule real-time (SMRT) sequencing comes of age: applications and utilities for medical diagnostics. Nucleic Acids Res.

[34] Weirather JL, de Cesare M, Wang Y, Piazza P, Sebastiano V, Wang X-J, et al. Comprehensive comparison of Pacific Biosciences and Oxford Nanopore Technologies and their applications to transcriptome analysis. F1000Research. 2017;6. 10.12688/f1000research.10571.1 PMC5553090 28868132

[35] Weston S, Frieman MB. COVID-19: Knowns, Unknowns, and Questions. mSphere. 2020;5(2). 10.1128/mSphere.00203-20 PMC7082143 32188753

[36] illumina. Advantages of paired-end and single-read sequencing 2021 [updated 2021. Available from: https://www.illumina.com/science/technology/next-generation-sequencing/plan-experiments/paired-end-vs-single-read.html.

[37] Rhoads A, Au KF (2015). PacBio sequencing and its applications. Genomics Proteomics Bioinformatics.

[38] Tegally H, Wilkinson E, Giovanetti M, Iranzadeh A, Fonseca V, Giandhari J (2021). Detection of a SARS-CoV-2 variant of concern in South Africa. Nature.

[39] Morel B, Barbera P, Czech L, Bettisworth B, Hübner L, Lutteropp S, et al. Phylogenetic analysis of SARS-CoV-2 data is difficult. bioRxiv. 2020:2020.08.05.239046. 10.1093/molbev/msaa314 PMC7798910 33316067

[40] Rambaut A, Holmes EC, O'Toole A, Hill V, McCrone JT, Ruis C (2020). A dynamic nomenclature proposal for SARS-CoV-2 lineages to assist genomic epidemiology. Nat Microbiol.

[41] Abernathy E, Glaunsinger B (2015). Emerging roles for RNA degradation in viral replication and antiviral defense. Virology.

[42] Quick J. nCoV-2019 sequencing protocol. Protocols io [Google Scholar]. 2020.

[43] Cleemput S, Dumon W, Fonseca V, Abdool Karim W, Giovanetti M, Alcantara LC (2020). Genome detective coronavirus typing tool for rapid identification and characterization of novel coronavirus genomes. Bioinformatics.

[44] Bolger AM, Lohse M, Usadel B (2014). Trimmomatic: a flexible trimmer for Illumina sequence data. Bioinformatics.

[45] Buchfink B, Xie C, Huson DH (2015). Fast and sensitive protein alignment using DIAMOND. Nat Methods.

[46] Bankevich A, Nurk S, Antipov D, Gurevich AA, Dvorkin M, Kulikov AS (2012). SPAdes: a new genome assembly algorithm and its applications to single-cell sequencing. J Comput Biol.

[47] Nick Loman WR, Andrew Rambaut. nCoV-2019 novel coronavirus bioinformatics protocol 2020-01-23 [Available from: https://artic.network/ncov-2019/ncov2019-bioinformatics-sop.html.

[48] Aksamentov I, Roemer C, Hodcroft E, Neher R (2021). Nextclade: clade assignment, mutation calling and quality control for viral genomes. J Open Source Softw.

[49] Nguyen LT, Schmidt HA, von Haeseler A, Minh BQ (2015). IQ-TREE: a fast and effective stochastic algorithm for estimating maximum-likelihood phylogenies. Mol Biol Evol.

